# Investigating Crystallization and Morphology of PLLA/PTMC Triblock Copolymer Solid Electrolytes

**DOI:** 10.1021/acs.macromol.5c02216

**Published:** 2025-10-30

**Authors:** Adriana Saldívar-Martínez, Monika Król, Janne Ruokolainen, Tim Melander Bowden

**Affiliations:** † Department of Chemistry—Ångström Laboratory, Division of Macromolecular Chemistry, 8097 Uppsala University, Uppsala Box 538, Sweden; ‡ Department of Applied Physics, School of Science, Aalto University, Espoo, Finland 00076, Finland

## Abstract

ABA-type block copolymers (BCPs) of poly­(trimethylene carbonate-*co*-trimethylene ether) (PTMC-*co*-PTME) and poly-l-lactic acid (PLLA) were synthesized through ring-opening polymerization. The BCP was blended with varying concentrations from 10 to 30 wt % lithium bis­(trifluoro methylsulfonate) (LiTFSI) to form solid polymer electrolytes (SPEs). Electrochemical impedance spectroscopy was used to study the ionic conductivity of the SPEs in the temperature interval from 30 to 150 °C. Simultaneous small-angle X-ray scattering and wide-angle X-ray scattering were used to study the kinetics of crystallization, together with the phase behavior of those BCPs. It was found that PLLA-*b*-PTMC-*co*-PTME-*b*-PLLA exhibits hierarchical organization at the micro- and nanoscale. More specifically, ABA BCP formed spherulitic superstructures, composed of alternating layers of primarily α^′^-form crystalline PLLA and amorphous regions. Interestingly, the amorphous phase consisted of phase-separated nanodomains of PLLA, embedded in the PTMC-*co*-PTME matrix. The investigation into the interplay between crystallization and phase separation via time-resolved scattering methods revealed that the crystallization of PLLA is the driving force for self-assembly at two distinct scales. The microstructure was similar, independent of the salt content; however, elevated doping of LiTFSI slowed the crystallization rate of PLLA and affected the crystalline phase composition. The presence of a crystalline phase slightly lowers the observed ionic conductivity. The best-performing electrolyte, with 20 wt % of LiTFSI, showed a conductivity of 1.2 × 10^–6^ S cm^–1^ at 60 °C.

## Graphical abstract

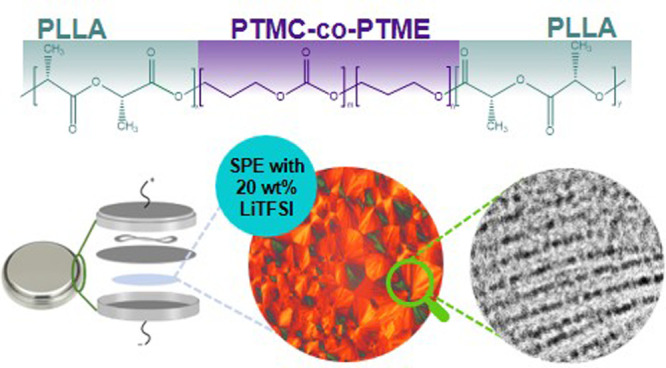

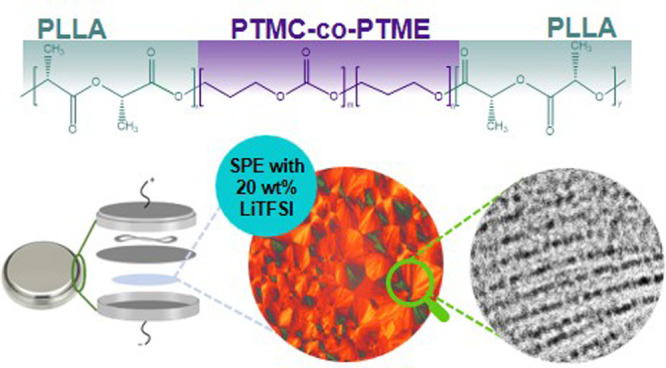

## 1 Introduction

The most widely investigated solid polymer electrolytes (SPEs) incorporate derivatives of semicrystalline poly­(ethylene oxide) (PEO), which act as a host for lithium salts.^1^ Despite its low glass-transition temperature (*T*_g_) (approximately −60 °C) and strong ability to solvate lithium salts,^2^ PEO-based systems still face critical challenges that need to be addressed. Namely, intrinsic high crystallinity hinders its application in the battery systems operating below PEO’s melting temperature (*T*_m_ = 66–75 °C),^3^ as the majority of ion conduction is carried out in the amorphous phase.^4^ On the other hand, reduced crystallinity deteriorates PEO’s mechanical strength at above *T*_m_. It substantially limits the use of PEO-based electrolytes in high-temperature battery setups, as poor strength modulus may result in dendrite formation.^5^ It is also worth noting that the properties of the polymeric electrolyte also depend on different cation-coordinating units present in the SPE. For PEO, lithium ions are transported primarily by interaction with ether groups, where Li–O bonds are formed and subsequently broken. The coordination and complexation strength of ether groups toward lithium ions is relatively high, which effectively traps them, rendering a low lithium transference number (*T*_Li+_ = 0.2).^6^ ^,^ ^7^ This is problematic as it may lead to polarization of the cell and also indicates that most of the ionic conductivity does not originate from the movement of the cation.^7^

Therefore, alternative SPE chemistries are being heavily investigated to tackle said challenges: high crystallinity degree of the conductive phase and poor cation transference number. Poly­(trimethylene carbonate) (PTMC) is a fully amorphous polymer with *T*_g_ = −16 °C, of relatively low ionic conductivity,^8^ but with excellent cation transport properties (*T*_Li+_ = 0.8).^9^ This is due to the presence of the carbonyl groups, which have moderate coordination and complexation strength of the lithium cation.^10^ One strategy to increase the conductivity of PTMC involves increasing the flexibility of its constituent polymer chains by lowering its *T*_g_. PTMC is not as molecularly flexible as PEO, thus limiting the ionic conductivity of PTMC-based electrolytes and their applicability as SPEs.

Mindemark et al.^11^ have shown that copolymerizing two monomers with different ion-coordinating groups may be a feasible route to address the low conductivity of the respective homopolymers. The authors synthesized a series of PTMC-based copolymers containing varying molar fractions of ε-caprolactone (caprolactone (CL)) units. The introduction of CL in the structure resulted in decreased *T*_g_ of the electrolytes in comparison to pure PTMC and hence an increase in ionic conductivity. In a follow-up study,^12^ TMC units were incorporated into the PCL backbone. This not only suppressed crystallinity in the polyester material but also gave higher ionic conductivity in the temperature range exceeding the melting point of the PCL crystallites.

Following this approach, poly­(trimethylene carbonate-*co*-trimethylene ether) (PTMC-*co*-PTME) is an interesting salt host choice as it incorporates two functionalities by having both carbonyl and ether groups. It takes advantage of the properties of PTMC in terms of *T*_Li+_, while exhibiting enhanced ionic conductivity due to the lowered *T*_g_ through the addition of ether-containing repeating units.

One route to achieving high modulus in the SPE is to couple a conductive block with a thermodynamically incompatible rigid block characterized by high *T*_g_, thus obtaining phase-separated block copolymer (BCP) electrolytes that combine the need for electrochemical and mechanical properties. Semicrystalline poly-l-lactic acid (PLLA) serves as an attractive choice for the rigid block of BCP SPEs as it can be obtained from renewable sources, and it is fully biodegradable.^13^ The high melting temperature of PLLA provides an opportunity to widen the operational temperature of SPEs and can be used in elevated temperature battery setups. Olmedo-Martínez et al.^14^ have shown that blending PEO with PLLA and LiTFSI provides the needed mechanical strength to design SPEs operating above 70 °C. They attributed this to the presence of PLLA crystals, reinforcing the film structure and having a relatively high melting temperature. Additionally, it was demonstrated by Xie et al.^15^ that LiTFSI addition (0.5–5 wt % with respect to PLLA) can act as a nucleation agent promoting PLLA crystallization. This finding may have interesting implications in the context of electrolyte systems as LiTFSI is the main doping agent. Furthermore, these previous studies explored different strategies to adjust a specific property, such as lowering the *T*_g_, or suppressing crystallinity to increase conductivity, or blending with a crystalline material to enhance the mechanical properties. Although the subject of crystallization in block copolymers has attracted much attention, more complex scenarios with the addition of salt and a detailed study of the crystallization kinetics and morphology of both blocks in terms of SPEs have not been reported, to the best of our knowledge.

The higher crystalline content of PLLA results in stronger, however less ductile material, compared to its more amorphous counterpart.^16^ PLLA is polymorphic, which means that its different crystallographic forms can be grown depending on the thermal treatment or the casting route.^17^ The most stable and prevalent form, α, is obtained by crystallizing PLLA above 120 °C. In the intermediate 100–120 °C range, a mix of α^′^ and α phases is present. Finally, the pure α^′^ phase is grown in a temperature regime below 90 °C. α^′^-Crystals are metastable, characterized by a bigger lattice dimension and a more disordered structure in comparison to the α phase.^18,19^ Varying structural forms of PLLA affect the material properties due to differences in the density of packing. Notably, Cocca et al.^20^ reported that films containing α-crystals have higher Young’s modulus in comparison to α^′^. The melting temperature of 100% crystalline PLLA is influenced by the crystalline form of PLLA, with α-crystals melting at around 180 °C and α^′^-crystals melting at around 150 °C^21^.

Merging the amorphous block with the semicrystalline counterpart can provide additional means to manipulate the self-assembly of the system via crystallization.^22–24^ In single-crystalline diblock systems, two mechanisms compete: phase separation and crystallization.^25^ This leads to two distinct morphology types upon cooling: breakout crystallization and confined crystallization. The mode of crystallization is strongly dependent on the degree of microphase segregation of BCP in the melt state. The breakout crystallization typically occurs in weakly segregated systems, whereas confined crystallization is prevalent in strongly segregated ones. These are also closely linked to the characteristics of the BCP’s system transition temperatures: *T*_g_ of the amorphous block, the crystallization temperature of the semicrystalline block (*T*_C_), and the order-to-disorder temperature of the BCP (*T*_ODT_). In the breakout crystallization (occurring when *T*_C_ > *T*_ODT_), the initial melt morphology is destroyed. Upon crystallization of the system, it is replaced by a lamellar (LAM) structure, where crystalline phases alternate with amorphous regions. If crystallization takes place from a fully mixed melt, the aforementioned LAM morphology is observed as well.^26^ On the other hand, the confined crystallization (occurring when *T*_ODT_ > *T*_g_ > *T*_C_) takes place within the pre-existing, microphase-separated domains of the BCP melt.

In this study, PLLA was added to the ion-conducting PTMC-*co*-PTME to act as a mechanical reinforcement, obtaining a PLLA-*b*-PTMC-*co*-PTME-*b*-PLLA triblock copolymer (Scheme 1). The prepared BCPs were doped with varying concentrations (0–30 wt %) of lithium bis­(trifluoromethanesulfonyl)­imide (LiTFSI). The PLLA crystalline phase in the SPEs potentially optimizes the mechanical properties and ionic conductivity by promoting phase separation. Additionally, PLLA’s high *T*_m_ allows for a wider range of operating temperatures for batteries. The electrolytes were subjected to thermal, morphological, and electrochemical investigations.

**Scheme 1:**
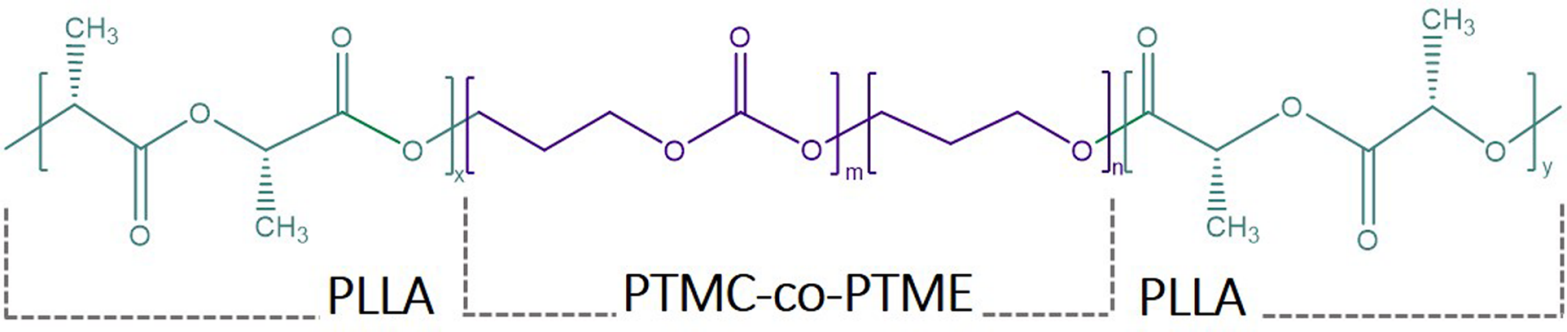
Structure of PLLA-*b*-PTMC-*co*-PTME-*b*-PLLA BCP

## 2 Experimental Section

### 2.1 Materials

Trimethylene carbonate (TMC, Richman Chemicals), diphenylammonium triflate (DPAT, Sigma), basic aluminum oxide (Al_2_O_3_, Sigma), tetrahydrofuran (THF, Sigma), methanol (Sigma), deuterated chloroform (CDCl_3_, Sigma), l-lactide (L-LA) (Sigma), stannous octoate (Sn­(Oct)_2_, Sigma), and toluene (Sigma) were used as received from the supplier. Lithium bis­(trifluoromethylsulfonyl)­imide (LiTFSI, BASF) was dried at 120 °C for 24 h under vacuum before use.

### 2.2 Polymer Synthesis of Macroinitiator (PTMC-*co*-PTME Block)

The monomer (TMC, 2 g, 19.59 mmol) and catalyst (DPAT, 0.6 mg, 0.001959 mmol) were added to a predried 50 mL Schlenk flask. The flask was sealed with a silicone septum, degassed, and left under a N_2_ gas flow. The flask was then placed in an oil bath at 60 °C for 48 h, and the temperature was subsequently raised to 120 °C for 24 h. The system was quenched with THF and filtered through basic Al_2_O_3._ The solvent was left to evaporate, and the final product was dried in a vacuum oven. *M*_n_, gel permeation chromatography (GPC) = 18,680 g mol^–1^; PDI, GPC = 1.7.

### 2.3 Synthesis of PLLA-*b*-PTMC-*co*-PTME-*b*-PLLA Block Copolymer

0.66 g of the macroinitiator (PTMC-*co*-PTME, 0.0694 mmol), 69.4 μL of Sn­(Oct)_2_, 0.1 M in toluene (0.000694 mmol, 0.05% mol vs monomer), 2 mL of toluene, and 2 g of the monomer (13.88 mmol, L-LA) were added to a predried 50 mL Schlenk flask. The flask was sealed with a silicone septum and flushed with N_2_ gas. The flask was then placed in an oil bath at 110 °C for 72 h. At the end of the reaction, 4 mL of toluene was added to dissolve the product completely, which was then precipitated dropwise in 400 mL of cold methanol. Next, the product was filtered with a Buchner funnel and introduced into a vacuum oven to dry. *M*_n_, ^1^H nuclear magnetic resonance (NMR) = 68,350 g mol^–1^; PDI, GPC = 1.57.

### 2.4 Electrolyte Preparation

To prepare the polymer electrolyte films, PLLA-*b*-PTMC-*co*-PTME-*b*-PLLA and LiTFSI were dissolved in THF. For the total electrolyte mass, three salt content concentrations were used: 10, 20, and 30 wt %. The solutions were placed in PTFE molds and dried by controlled solvent evaporation. To prepare the electrolyte films, the previously cast material was pressed at room temperature with a pressure of 20–30 MPa for 5 min, with Teflon rings used to obtain disks of the desired diameter. The whole process was performed inside an Ar-filled glovebox. Disks were obtained with a thickness of 250–300 μm, which were averages based on at least five different measurements using a digital indicator (Mitutoyo).

### 2.5 Characterization Methods

#### 2.5.1 ^1^H Nuclear Magnetic Resonance

^1^H NMR spectra were recorded with CDCl_3_ on a JEOL Eclipse+ 400 MHz spectrometer.

#### 2.5.2 Gel Permeation Chromatography

An Agilent 1260 Infinity GPC instrument was used to measure the molecular weights and polydispersity index (PDI) (*M*_w_/*M*_n_) of the polymer samples. The GPC instrument was fitted with PolyPore columns and an RI detector. The mobile phase was THF (1 mL min^–1^), and the analysis was performed at 35 °C. PMMA standards were used to calibrate the system.

#### 2.5.3 Differential Scanning Calorimetry

Nonisothermal differential scanning calorimetry (DSC) tests were performed to characterize the electrolytes. A Mettler Toledo DSC 3+ with STARe software was used. A heating/cooling/heating cycle was used from −40 to 170 °C under a flow of N_2_ gas.

To fully remove the thermal history, the samples were first heated from −40 to 170 °C at a rate of 10 °C min^–1^ and maintained at this temperature for 2 min. The samples were then cooled to 100 °C at 5 °C min^–1^, where they underwent a 5 min annealing process. After annealing, the samples were further cooled to −40 °C at 5 °C min^–1^. A second heating scan up to 170 °C at 10 °C min^–1^ was then carried out, and a second cooling procedure was performed as previously described but with the annealing duration extended to 30 min. The heating and cooling processes were repeated in a loop but with the annealing time increased in each case to include 1 h, 3 h, 5 h, 7 h, 10 h, 12 h, and, in some cases, up to 24 h.

#### 2.5.4 Small-Angle X-ray Scattering and Wide-Angle X-ray Scattering

Simultaneous temperature-resolved small-angle X-ray scattering (SAXS) and wide-angle X-ray scattering (WAXS) measurements were recorded via Xenocs XEUSS 3.0. equipped with Dectris 1 M hybrid pixel and Dectris Eiger 500k detectors, respectively. The experiment was conducted utilizing a Cu Kα radiation source with λ = 1.54 Å. Both detectors were calibrated with LaB_6_. The measurement chamber was evacuated to limit parasitic scattering from the air.

One-dimensional scattering intensity profiles were plotted as a function of scattering vector, *I*(*q*), or scattering angle, *I*(2θ), by integrating the collected 2D detector data via XSACT software. Scattering vector *q* was given by *q* = 4π·sinθ/λ, where 2θ is the scattering angle. SAXS spectra were recorded covering a range corresponding to *q* = 0.1–1 nm^–1^ and WAXS 2θ = 10–25°. The data were subjected to standardized normalization protocols.

The samples were loaded between two Kapton foils into the heating stage connected to an INSTEC mK2000 temperature control unit. Pristine (no salt) and salt-doped BCPs were heated to 170 °C at 5 °C/min and kept at this temperature for approximately 5 min to erase the previous thermal history. In the next step, samples were gradually cooled to 5 °C/min and crystallized at 100 °C. For the first hour, the spectra were acquired every minute to resolve the interplay between crystallization and phase separation. The sampling frequency was consecutively decreased, with SAXS/WAXS scattering intensities acquired after 3, 7, and 12 h and finally after 24 h. Lastly, all samples were cooled to room temperature to acquire the final scattering spectra. WAXS spectra were normalized by their sum. The crystalline fraction was estimated using the XSACT software.

#### 2.5.5 Transmission Electron Microscopy

The annealed polymer samples, retrieved from SAXS measurements, were mounted to the pin and microtomed on Leica EM UC7 at −40 °C via a Diatome 35° diamond knife. The polymer ribbons were deposited on a 300 mesh copper grid coated with lacey carbon, plunged into liquid nitrogen, and heated to room temperature under vacuum to prevent water-imposed changes to the microstructure. Cryo-transmission electron microscopy (cryo-TEM) characterization was conducted on either a JEOL JEM-3200FSC (300 kV) or Tecnai 12 (120 kV). In the case of cryo-TEM (JEOL JEM-3200FSC), the temperature of the examined specimen was kept at −174 °C during the imaging process. For Tecnai 12, a special cryo-holder was utilized, where the temperature of the examined specimen was kept at −178 °C.

The micrographs were recorded in bright-field mode under slight underfocus. Additionally, for JEOL JEM-3200FSC acquisitions, a zero-energy loss omega-type filter (slit width 30 eV) was utilized. Pictures were recorded via a Gatan Ultrascan 4000 CCD (JEOL JEM-3200FSC) or Gatan Ultrascan 1000 CCD (Tecnai 12).

#### 2.5.6 Polarized Light Optical Microscopy

Pristine and salt-doped BCPs were dissolved in THF, passed through a Nylon66 pore size 0.45 μm filter, and dried under vacuum overnight. The recovered powder was placed between two glass slides and melted at 170 °C in a vacuum oven to minimize the formation of air bubbles.

Nonisothermal crystallization behavior and the spherulite growth rate were analyzed under cross-polarized filters on a Leica 1090 optical microscope equipped with a Leica MC190 HD camera and Linkam heating stage. Previously prepared slides were heated to 170 °C and kept at this temperature for 5 min until the complete disappearance of the formed superstructures was observed. Subsequently, the BCPs were cooled to 100 °C at a rate of 5 °C/min. The micrographs were recorded as time-lapses for the given crystallization time.

The spherulitic growth rate was estimated by tracking the radius of spherulites as a function of time using ImageJ software.

#### 2.5.7 Ionic Conductivity

The SPEs were sandwiched between two stainless steel blocking electrodes in CR2032-type coin cells inside an Ar-filled glovebox. The impedance response was recorded for frequencies 1–10 MHz at a 10 mV amplitude using a Schlumberger SI 1260 Impedance/Gain-phase Analyzer instrument. Before measurements, the coin cells were heated to 150 °C for 10 min and then annealed at 100 °C for 24 h. After annealing, the cells were cooled to room temperature. The measurements were carried out at room temperature and every 10 °C interval between 30 and 150 °C using a precision hot plate and allowing the coin cells to heat for at least 30 min at each temperature point or until stability was reached prior to each measurement. Conductivity was calculated by using the bulk resistance.

## 3 Results and Discussion

A two-step procedure was carried out to synthesize the BCP. The PTMC-*co*-PTME macroinitiator was synthesized via bulk ring-opening polymerization (ROP) of just one monomer (TMC). By using the metal-free catalyst DPAT, a PTMC-*co*-PTME random copolymer was obtained with 9 mol % of ether content, confirmed by ^1^H NMR, Figure S1 available in the Supporting Information. In the second step, after purification and drying, L-LA and the traditionally used catalyst Sn­(Oct)_2_ were added to produce the PLLA blocks. The utilized catalyst must be able to restrict the side reactions generally occurring in the ROP of lactide (LA), especially when L-LA is used to generate stereoregular PLLA, which is particularly important to promote crystallization.^27^ The absence of more complex signals in the 1^H^ NMR spectrum (Figure 1) shows a good retention of the stereoregularity. If we consider the molecular weight for the PLLA repeat unit to be 144 g/mol, the block composition in the copolymer is thus 34.7 wt % PTMC-*co*-PTME and 65.3 wt % PLLA with *M*_n_ of 68,350 g mol^–1^.

**Figure 1:**
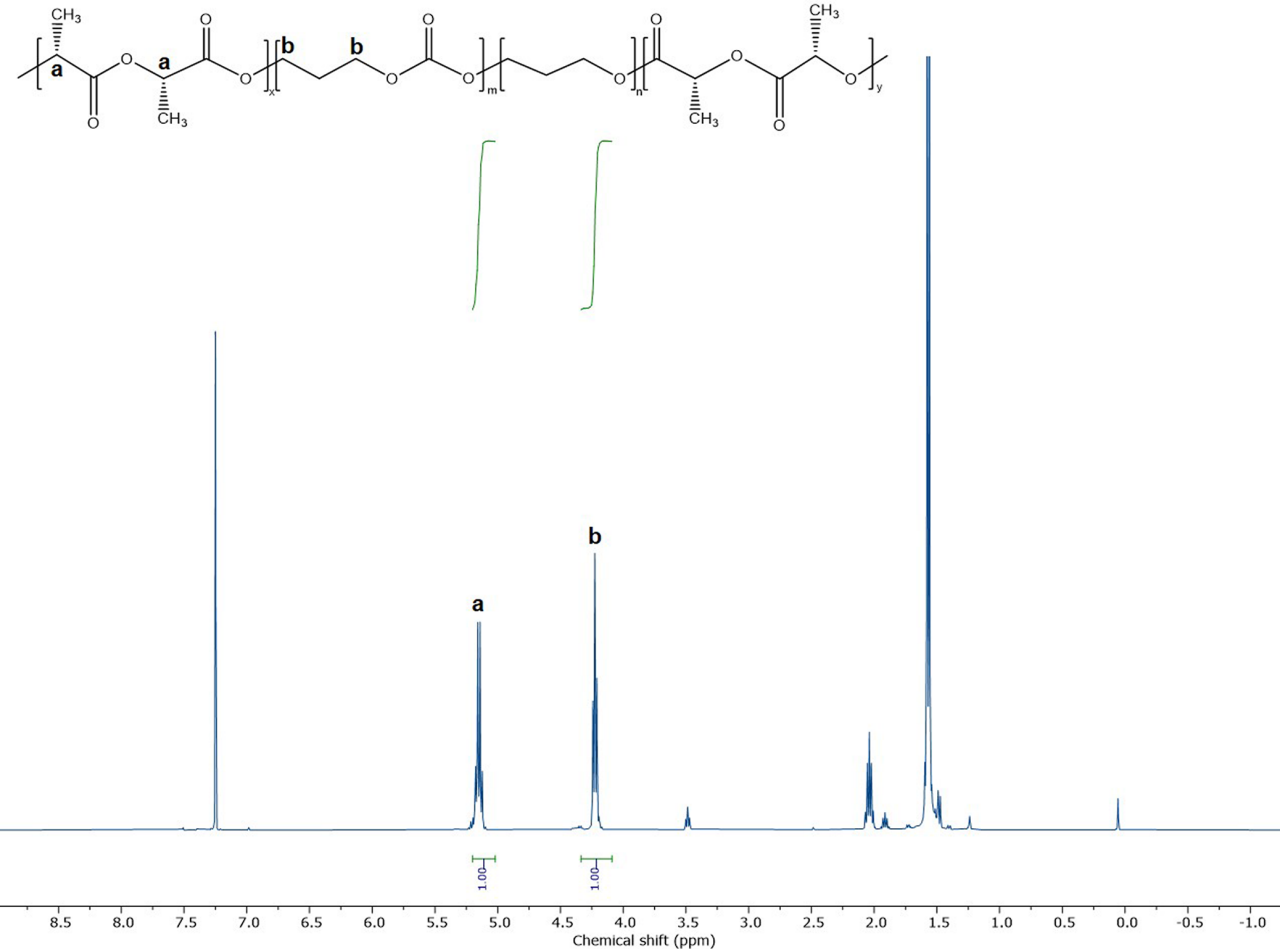
^1^H NMR spectrum of PLLA-b-PTMC-co-PTME-b-PLLA in CDCl_3_.

### 3.1 Nonisothermal DSC of PLLA-*b*-PTMC-*co*-PTME-*b*-PLLA Electrolytes

Figure 2 shows the nonisothermal DSC scans for the PTMC-*co*-PTME, PLLA-*b*-PTMC-*co*-PTME-*b*-PLLA BCP, and the polymer electrolytes with different salt contents.

**Figure 2:**
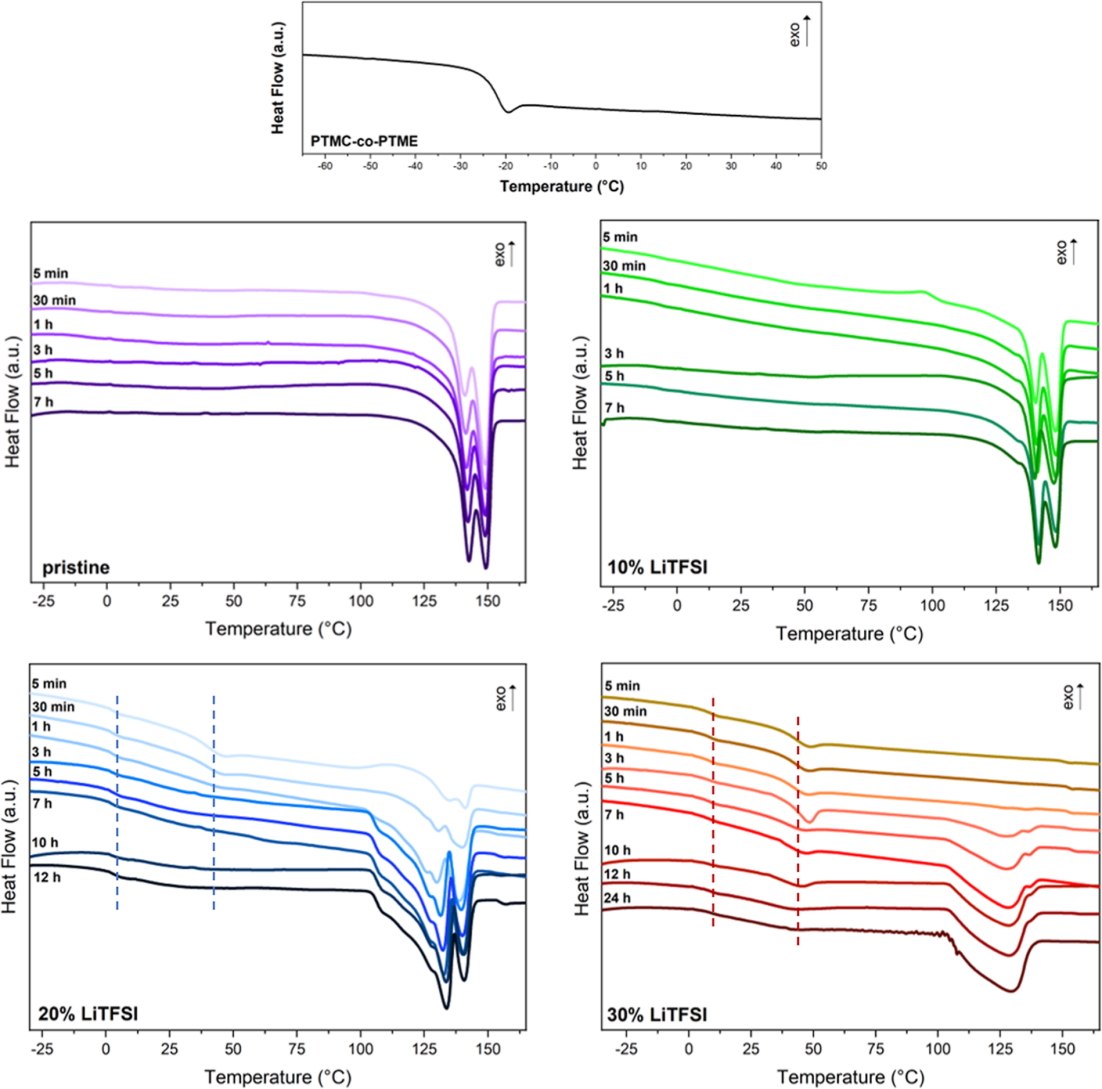
DSC of PTMC-co-PTME and PLLA-b-PTMC-co-PTME-b-PLLA electrolytes with varying LiTFSI contents and different annealing times.

In Figure 2, we observe that all compositions of BCPs present an endothermic peak that corresponds to the melting of pure PLLA crystallites, indicating a PLLA-rich phase. This confirms that after very short annealing times at 100 °C, PLLA crystals are always present in all electrolyte compositions, except for the one containing 30 wt % LiTFSI, which requires at least 5 h of annealing. During the second heating scan of the pristine PLLA-*b*-PTMC-*co*-PTME-*b*-PLLA copolymer, *T*_m_ of the PLLA-rich phase is observed at 124–154 °C. The appearance of two melting peaks in this range suggests the presence of different crystallite populations of PLLA,^17,28^ which is later explored in the SAXS/WAXS investigation. The *T*_m_ value is slightly lower than the reported melting temperatures for both α- and α′-crystals in the PLLA homopolymer.^21^ This depression in *T*_m_ is likely due to the presence of the PTMC-*co*-PTME block, which causes a diluent effect due to the partial miscibility of the different phases. Additionally, the thermal behavior of PLLA was found to be dependent on the LiTFSI content. In the copolymers with Li salt, the melting temperature of the PLLA phase further decreases with increasing salt concentration, as LiTFSI acts as a plasticizer. This indicates that some salt is present in the PLLA phase, as LiTFSI is thermodynamically miscible with PLLA,^15^ although the Li salt prefers the PTMC-*co*-PTME-rich phase, which is confirmed by the conductivity results discussed in the following sections.

The absence of *T*_g_ at around −23 °C, which corresponds to *T*_g_ of pure PTMC-*co*-PTME, suggests that it is partially miscible with PLLA. In the DSC curves for the copolymer compositions containing 20 and 30 wt % Li salt, we observe two *T*_g_ values (marked by the dotted lines), which indicate that the system has 3 phases: an amorphous PTMC-*co*-PTME-rich phase, an amorphous PLLA-rich phase, and a crystalline PLLA-rich phase. The first *T*_g_, at around 4 °C in the BCP with 20 wt % LiTFSI, corresponds to a mixed PTMC-*co*-PTME/PLLA phase, where *T*_g_ of the PTMC-*co*-PTME (−23 °C) block increases due to partial miscibility with amorphous PLLA, which has a higher *T*_g_ (60 °C).^14,15,29^ In the BCP with 30 wt % LiTFSI, the first *T*_g_ further increases by a few degrees to around 9 °C, likely due to the higher concentration of salt, which leads to the formation of cross-links between polymer chains and ions, thereby restricting chain movement and thus increasing *T*_g_.^30^ This is the same behavior observed for just PTMC-*co*-PTME with different salt loadings, as shown in the DSC curves available in the Supporting Information (Figure S2), where *T*_g_ of the copolymer increases with a higher salt content. The second *T*_g_ at around 43 °C is attributed to an amorphous-rich PLLA but with lower values than those previously reported for the PLLA homopolymer due to the presence of salt.

Overall, partial miscibility is evident from the changes in the *T*_g_ values when compared to those of the neat polymers, while also being affected by the presence of LiTFSI. Differences in *T*_g_ may also be due to distinct specifications of the synthesized BCPs such as, in particular, low molecular weight.

As the mechanical properties of polymers are directly related to the crystallinity degree,^16^ the presence of PLLA crystals implies that these electrolytes’ mechanical properties may be improved in contrast to electrolytes based on PTMC-*co*-PTME.

Figure 3 shows the calculated crystallinity degree of the BCP without salt and the polymer electrolytes with different salt contents, depending on the annealing time.

**Figure 3:**
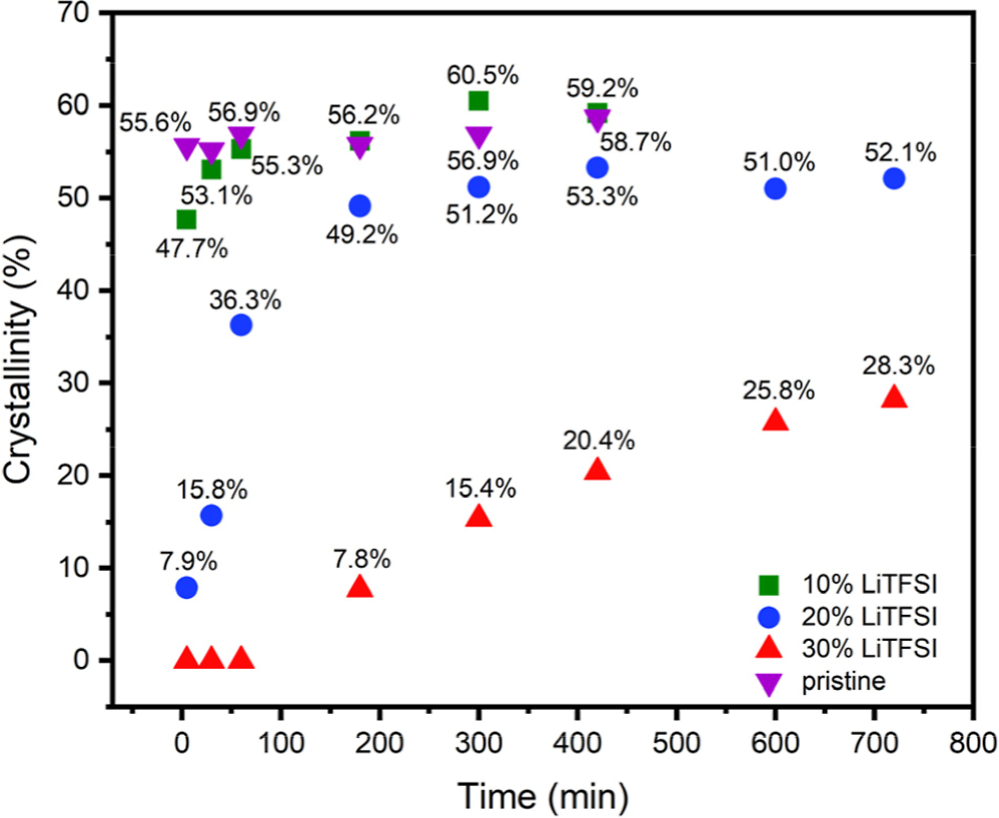
Crystallinity degree of the BCP without salt and with varying concentrations of LiTFSI with different annealing times at 100 °C.

The crystallinity degree of the PLLA phase was obtained by integrating the melting peaks into the DSC curves. The enthalpy of fusion (Δ*H*_f_) was then estimated by considering only the wt % of PLLA present in each sample. Finally, the crystallinity was calculated with the following equation:

$$X=\frac{\Delta H_{f}}{\Delta H_{f}^{{}^{\circ}}}$$

where Δ*H*_f_^°^ corresponds to the enthalpy of fusion of fully crystalline PLLA homopolymer, which is 93 J/g.^31^

The crystallization rates of the BCP without salt and the copolymer containing 10 wt % of LiTFSI are similar. However, the overall crystallization rate decreases as the salt concentration is increased. This indicates once more that LiTFSI is thermodynamically miscible with PLLA. It is generally accepted that the salt acts as a solvent that decreases both the crystallization degree and rate, and also the melting temperature of PLLA, by diluting the concentration of polymer chains.^15,32^

It is important to highlight that the composition of the BCP is 65.3 wt % of PLLA only when the BCP is pristine. Once the salt is added, and we are instead dealing with an electrolyte, note that there are two main phases: a conductive phase (soft block) and a nonconductive phase (hard block) comprised of crystalline PLLA. However, of the 65.3 wt % of PLLA available, only up to half of it can crystallize (depending on the salt content and annealing time), and the rest is found in the amorphous (conductive) phase. In other words, in the electrolyte with 20 wt % of LiTFSI, 52 wt % of the BCP corresponds to the hard block, and 48 wt % is the soft conductive block together with LiTFSI. Similarly, with 30 wt % of LiTFSI, 45.4 wt % of the BCP corresponds to the hard block, and the remaining 54.5 wt % is the soft conductive block together with LiTFSI.

### 3.2 Morphology

#### 3.2.1 Wide- and Small-Angle X-ray Scattering

The phase behavior of pristine and salt-doped PLLA-*b*-PTMC-*co*-PTME-*b*-PLLA was investigated by using temperature-resolved simultaneous WAXS and SAXS. All spectra are offset on the *y*-axis for clarity. On WAXS diffraction patterns, the dotted line represents the baseline of the graphs. The main objective was to trace the sequence of the structure formation and determine the nature of the self-assembly. The impact of LiTFSI doping was assessed based on its influence on the thermodynamic compatibility between constituent blocks and the influence on the crystalline fraction. The salt doping is known to (i) change the Flory–Huggins parameter, which signifies incompatibility between blocks,^33^ (ii) influence the volume ratios of the blocks assuming that one preferentially solvates LiTFSI, and (iii) affect the crystallization kinetics of PLLA.^15^

### 3.3 WAXS Analysis

Above 170 °C, the investigated systems were fully amorphous, as no characteristic peaks for PLLA crystals were detected in the WAXS spectra (Figure 4a–d).

**Figure 4:**
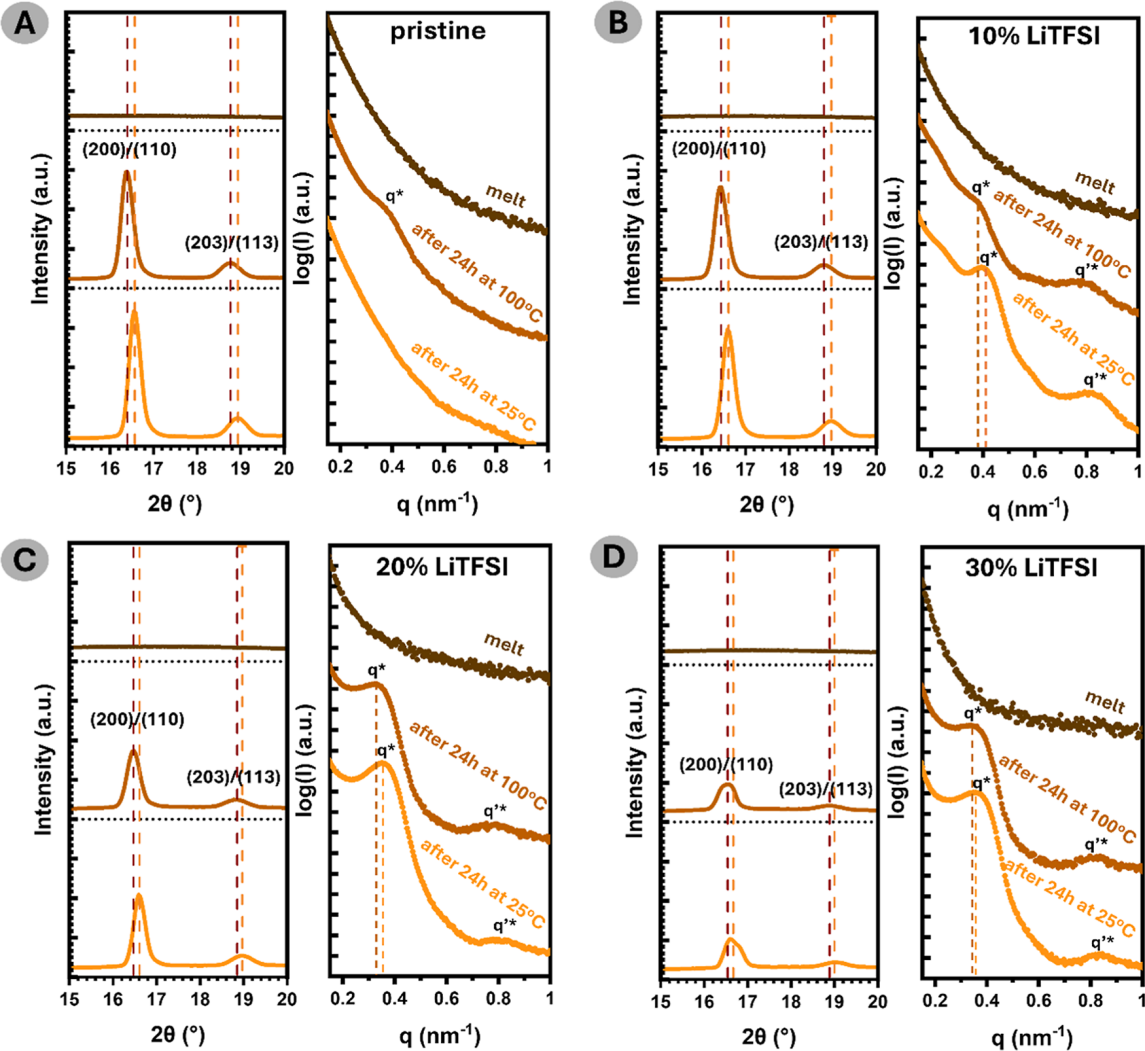
Temperature-dependent simultaneous WAXS–SAXS profiles of (a) pristine, (b) 10 wt % LiTFSI-doped; (c) 20 wt % LiTFSI-doped; (d) 30 wt % LiTFSI-doped PLLA-b-PTMC-co-PTME-b-PLLA.

As expected, the crystallization process was initiated after cooling the sample to 100 °C. First, the samples that were annealed for 24 h will be discussed. WAXS profiles depicting 2θ range between 10 and 30° are available in the Supporting Information (Figure S3).

The presence of the crystalline form of PLLA is confirmed by the diffraction patterns depicted in Figure 4a–d. At 100 °C, pronounced peaks belonging to (200,110) and (203,113) planes are located at 2θ ≈ 16.4° and 2θ ≈ 18.8°, respectively, whereas in the measurement performed after cooling the film to 25 °C, 2θ ≈ 16.6° and 2θ ≈ 19°. Two observations can be noted: (i) the possibility of thermal expansion of the lattice as peaks are displaced by 0.18° ± 0.02°, depending on the set temperature; and (ii) a small asymmetric shoulder at higher 2θ values of the peak reflecting the (200,110) planes, which indicates the presence of two convoluted bands.

The latter observation allows the theorization of the presence of two polymorphic forms of PLLA crystals, α′ and α. It is expected for samples crystallized at 100 °C, as observed previously in multiple studies.^28,34–36^ Figure 5a depicts a close-up of the peak belonging to (200,110) of all the films fitted with a Voigt function.^37^ This enabled the distinction of the α′ and α phase reflection contributions for both temperature regimes. 2θ values of α′(200,110) and α­(200,110) as a function of the salt concentration are depicted in Figure 5b. At 100 °C, the peaks representative of α′(200,110) and α­(200,110) are positioned at 16.4° ± 0.02° and 16.6° ± 0.02°, whereas at 25 °C, these are positioned at 16.6° ± 0.02° and 16.8° ± 0.04°.

**Figure 5:**
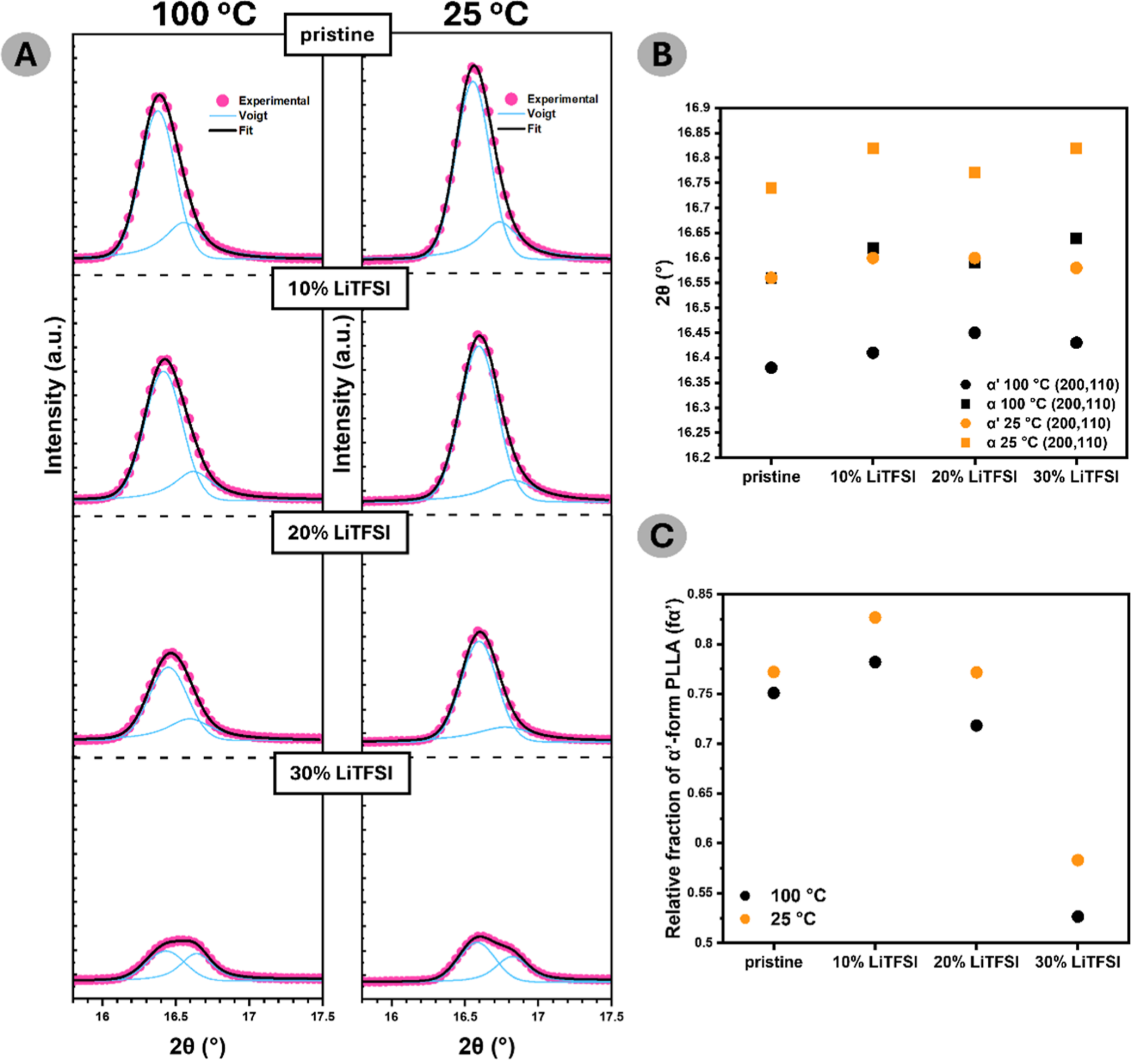
Examination of crystalline PLLA phase composition: (a) series of enlarged diffraction peaks corresponding to (200,110) planes for all sample compositions at two temperature points (100 and 25 °C). Experimental data are depicted as a pink scatter plot, cumulative Voigt fitting as a black line, and deconvoluted peaks as a blue line; (b) 2θ position of deconvoluted peaks, corresponding either to α or α′ form as a function of salt doping; (c) relative fraction of α′ phase as a function of salt doping.

Figure 5c represents the relative fraction of α′-form phase (*f*_α_^′^) depending on the LiTFSI concentration at two temperature points. It was obtained by dividing the integrated intensity of the deconvoluted α′ peak by the total integrated intensity of the (200,110) plane reflection, as shown previously. The majority of PLLA crystals belong to the α′ phase, independent of the sample composition, and α′-form content increases upon cooling the films down. However, it appears that the relative ratio of α′/α decreases with the critical value of salt doping, which can be seen by comparing the pristine film with 30% LiTFSI. A more thorough investigation is needed to explore this observation, which is not within the scope of this work.

The time-dependent crystallinity fraction (*f*_c_) of PLLA is listed in Table 1. As expected, the addition of salt significantly slowed crystallization kinetics, and this effect is more severe with the increased salt loading. The only remarkable exception is the sample blended with 10 wt % of LiTFSI, as at early annealing stages, the kinetics appear to be faster than in the pristine sample. This can possibly be attributed to the aforementioned positive effect of LiTFSI with regard to the nucleation density, which was also observed in the following POM investigation. Additionally, upon cooling the BCP series, the crystallized fraction increases, indicating secondary crystallization at lower temperatures. Most likely, this is an additional growth of PLLA in α′-form, for which nucleation has a lower energy barrier and is favored at temperatures below 100 °C. Note that the estimated crystallization fraction cannot be directly correlated to DSC data due to the slightly different thermal treatment of the samples.

**Table 1:** Crystallinity Fraction of PLLA (*f*_c_) Estimated at Different Annealing Times

	fraction of crystalline PLLA (*f*_c_)							
	*f*_c_ 5 min, 100 °C	*f*_c_ 30 min, 100 °C	*f*_c_ 1 h, 100 °C	*f*_c_ 3 h, 100 °C	*f*_c_ 7 h, 100 °C	*f*_c_ 12 h, 100 °C	*f*_c_ 24 h, 100 °C	*f*_c_ 24 h, 25 °C
pristine	0.31	0.33	0.35	0.46	0.49	0.48	0.49	0.64
10% LiTFSI	0.41	0.4	0.44	0.45	0.45	0.45	0.45	0.56
20% LiTFSI	0.13	0.33	0.35	0.32	0.31	0.34	0.34	0.44
30% LiTFSI	0	0	0.03	0.16	0.18	0.21	0.22	0.31

### 3.4 SAXS Analysis

In the melt state (Figure 4), no characteristic scattering maxima patterns were detected, indicating that the phases are fully mixed above 170 °C. This can be observed for all of the investigated compositions. When the samples were cooled down to the crystallization temperature, the scattering profiles revealed distinct structural peaks, signifying crystallization-prompted phase separation. In this process, the lamellar (LAM) microstructure is formed,^26^ consisting of alternating crystalline PLLA and amorphous PLLA/PTMC-*co*-PTME interlamellar regions.

For the pristine BCP, this morphology is confirmed by the presence of the long order peak at 0.4 nm^–1^ corresponding to the Bragg distance, *d* = 15.7 nm, denoted q* (refer to Figure 4a), following the gradual cooling and annealing at 100 °C. Interestingly, upon further cooling the structure to room temperature, any reminiscence of the phase separation disappears, based on the scattering intensity profile. This observation is attributed to temperature-induced changes in electron density contrast between crystalline lamellae and amorphous interlamellar regions, as well as/or possible alteration in the ratio of α′/α-crystals. As the density and composition of the crystalline phase change upon cooling, it is assumed that it ends up being closer to the one of the amorphous phase. Recently, Jariyavidyanont et al.^38^ discussed the correlation between different PLLA polymorphs (α and α′) and the resulting density contrast between crystalline and amorphous phases. Here, they directly showed that even though SAXS scattering patterns did not reveal the long LAM period for α′ PLLA, the long-range layering was still present, as evidenced by atomic force microscopy (AFM). They directly attributed this to the similar value of electron density of amorphous PLLA and α′-crystals. For α PLLA crystals, both the SAXS pattern and AFM confirmed the LAM microstructure. In our case, the amorphous phase is composed of PLLA and PTMC-*co*-PTME, where the resulting density could be closer to α′ PLLA.

To confirm that the formed microstructure was indeed still present, transmission electron microscopy (TEM) characterization of the pristine sample is described in further sections.

In samples doped with salt, the peak signifying the lamellar morphology q*, together with an additional peak, denoted *q*′*, can be observed, indicating the coexistence of the additional ordering at the smaller nanoscale. The presence of amorphous PLLA nanodomains in the interlamellar PTMC-*co*-PTME amorphous region was confirmed, corresponding to the magnitude of *q*′*. The following TEM investigation further elucidates the nature of the observed microstructure. First, the spectra recorded at room temperature will be discussed. For 10% LiTFSI, two peaks can be observed at q* = 0.42 nm^–1^ (*d* = 15 nm) and 0.83 nm^–1^ (*d*′ = 7.6 nm). At first glance, the peak at 0.83 nm^–1^ could be interpreted as the second-order reflection of LAM morphology, since its position closely follows the expected ratio of *q*/q* = 1:2:3···. However, taking into consideration the scattering patterns of all the salt-doped samples, two trends can be observed: upon increasing the salt content, the primary lamellar peak (q*) shifts to lower q values (indicating a larger lamellar spacing), while the second peak (*q*′*) remains at a nearly constant position (∼0.83 nm^–1^). This observation suggests that the 0.83 nm^–1^ peak observed for 10% LiFTSI is not a higher-order lamellar reflection but instead arises from the expelled amorphous PLLA nanodomains.

The salt doping has a primary influence on *d*-spacing, where the steepest change of the long period can be observed upon increasing the doping from 10 wt % (*d* = 15 nm) to 20 wt % (*d* = 17.4 nm). This can possibly be attributed to swelling of the amorphous phase due to LiTFSI solvation. For 20% LiTFSI, *q*′* was observed at 0.83 nm^–1^, corresponding to the Bragg distance of 7.6 nm. On the other hand, for 30% LiTFSI, q* was identified at 0.36 nm^–1^ and *q*′* at 0.83 nm^–1^, rendering *d* = 17.4 nm and *d* ^′^ = 7.6 nm, respectively.

Comparing the scattering intensity profiles obtained after 24 h of crystallization at the elevated temperature, with spectra taken after cooling to room temperature, reveals significant thermal expansion and contraction of the BCP lamellar domains. This is evident in the changes to the *d*-spacing, which decreases upon cooling by 1.5 nm for 10% LiTFSI. A similar dependency can be observed with an increased doping of salt (decrease by 1.4 and 0.5 nm for 20 and 30 wt % respectively). This is an interesting point considering the temperature-dependent operational window of the electrolyte, where a similar behavior can be expected. However, the direct impact of the dimensional changes of the microstructure induced by the temperature was not observed in the following conductivity measurement. Table 2 lists the temperature-dependent positions of the structural peaks and the corresponding *d*-spacings.

**Table 2:** List of Peak Positions and the Corresponding Characteristic Lengths

	q*,^100 °C^ (nm^–1^)	q*,^25 °C^ (nm^–1^)	*d*,^100 °C^ (nm)	*d*,^25 °C^ (nm)	*q*′*,^100 °C^ (nm^–1^)	*q*′*,^25 °C^ (nm^–1^)	*d*′,^100 °C^ (nm)	*d*′, ^25 °C^ (nm)
pristine	0.40		15.7					
10% LiTFSI	0.38	0.42	16.5	15	0.83	0.83	7.6	7.6
20% LiTFSI	0.33	0.36	18.8	17.4	0.83	0.83	7.6	7.6
30% LiTFSI	0.35	0.36	17.9	17.4	0.83	0.83	7.6	7.6

The study of the crystallization kinetics conducted on 20% LiTFSI (Figure 6) via time-resolved SAXS–WAXS reveals that phase separation is preceded by crystallization. A similar dependency can be observed for all compositions of the samples (Figures S4–S6). A slight uptake in the lower *q* region (Figure 6a), corresponding to the formation of lamellar regions due to the phase separation of crystalline PLLA lamellas from the amorphous matrix, is already visible after 5 min of the annealing process at 100 °C. WAXS spectra (Figure 6b) indicate that this is simultaneous with the PLLA crystalline phase formation, as the small peak at 2θ = 16.4° can be observed at the same time point.

**Figure 6:**
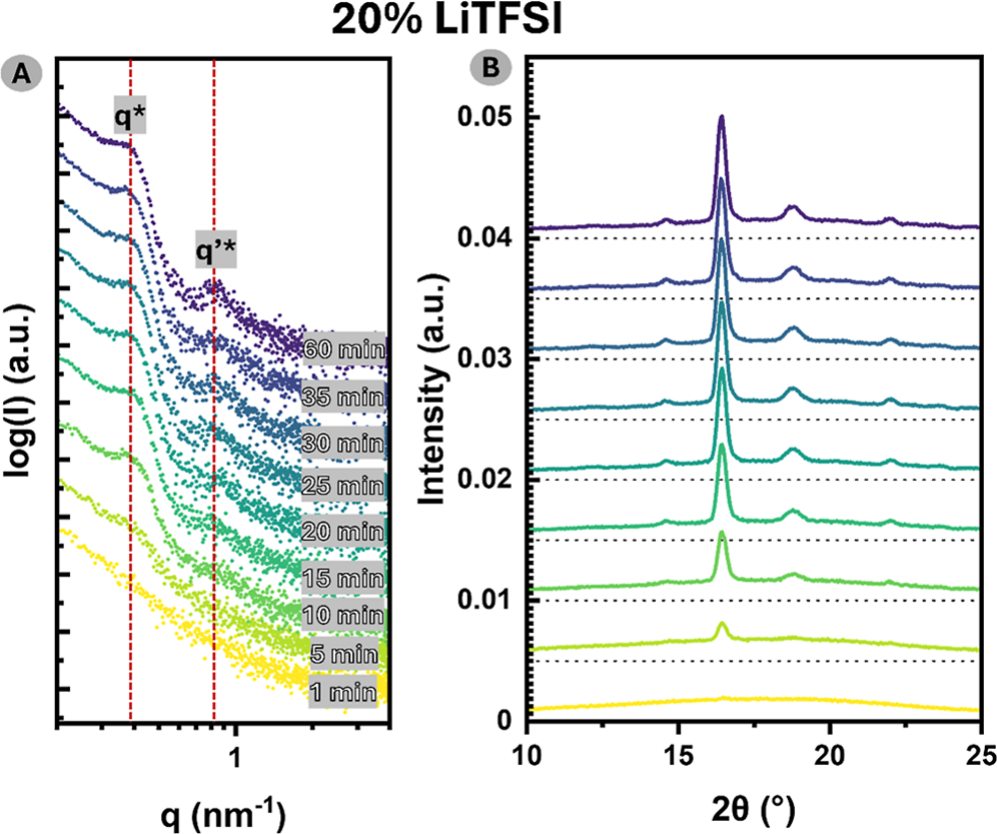
Time-dependent simultaneous SAXS–WAXS spectra for PLLA-*b*-PTMC-*co*-PTME-*b*-PLLA doped with 20 wt % of LiTFSI. The sample was nonisothermally crystallized at 100 °C. Timestamps are indicated on the figure.

This is followed by the appearance of the secondary peak at around the 15 min crystallization time point, corresponding to phase separation of amorphous PLLA within the PTMC-*co*-PTME phase. Upon further crystallization of the sample, structural peaks become proportionally more pronounced, indicating a stronger ordering of the microstructure. The phase separation of amorphous PLLA, prompted by crystallization of the block, can be explained by the change of its volume fraction with respect to amorphous PTMC-*co*-PTME. A similar morphology has been reported for a highly asymmetric double-crystalline PCL-*b*-PEO system.^39^

### 3.5 Transmission Electron Microscopy

Complementary TEM analysis provided more insights into the microstructure ordering. A microscopic investigation was performed on samples retrieved from SAXS after 24 h of crystallization at 100 °C, followed by cooling to room temperature. BCP sections were exposed to RuO_4_ vapor, which preferentially stains amorphous regions via diffusion. Since there are multiple amorphous components present (PTMC-*co*-PTME and PLLA), an additional investigation was conducted to determine the preferential sites for the stain. Phase-separated blends of PTMC-*co*-PTME and PLLA homopolymers were prepared and exposed to the stain. In Figure S7, illustrating the morphology of the PTMC-*co*-PTME:PLLA 4:1 blend, it is shown that RuO_4_ favors amorphous PLLA over PTMC-*co*-PTME in a given time frame (10 min). Hence, it is concluded that the darkest (most stain-saturated) phase is amorphous PLLA.

Coupling TEM results with previously discussed SAXS characterization, it is possible to deduce that lamellar morphology stems from the segregation of crystalline PLLA from a mixed amorphous PLLA/PTMC-*co*-PTME matrix. Simultaneously, it prompts the amorphous PLLA to phase-separate from PTMC-*co*-PTME in the form of nanodomains. The alternating lamellas of crystalline PLLA and amorphous nanodomains of PLLA embedded within the PTMC-*co*-PTME interlamellar region are well depicted in Figure 7a. Such morphology is consistently observed along all electrolyte compositions, independent of the salt content, further supporting that the phase separation of amorphous PLLA is not triggered by LiTFSI doping but by the crystallization of PLLA. The estimation of the spacing between phase-separated nanodomains of amorphous PLLA is shown in Figure 7b (*d*′ = 7.28 nm), which is in line with the magnitude of *q*′* identified by SAXS characterization. Finally, a scheme depicting the observed hierarchical microstructure at the nanoscale is shown in Figure 7c.

**Figure 7:**
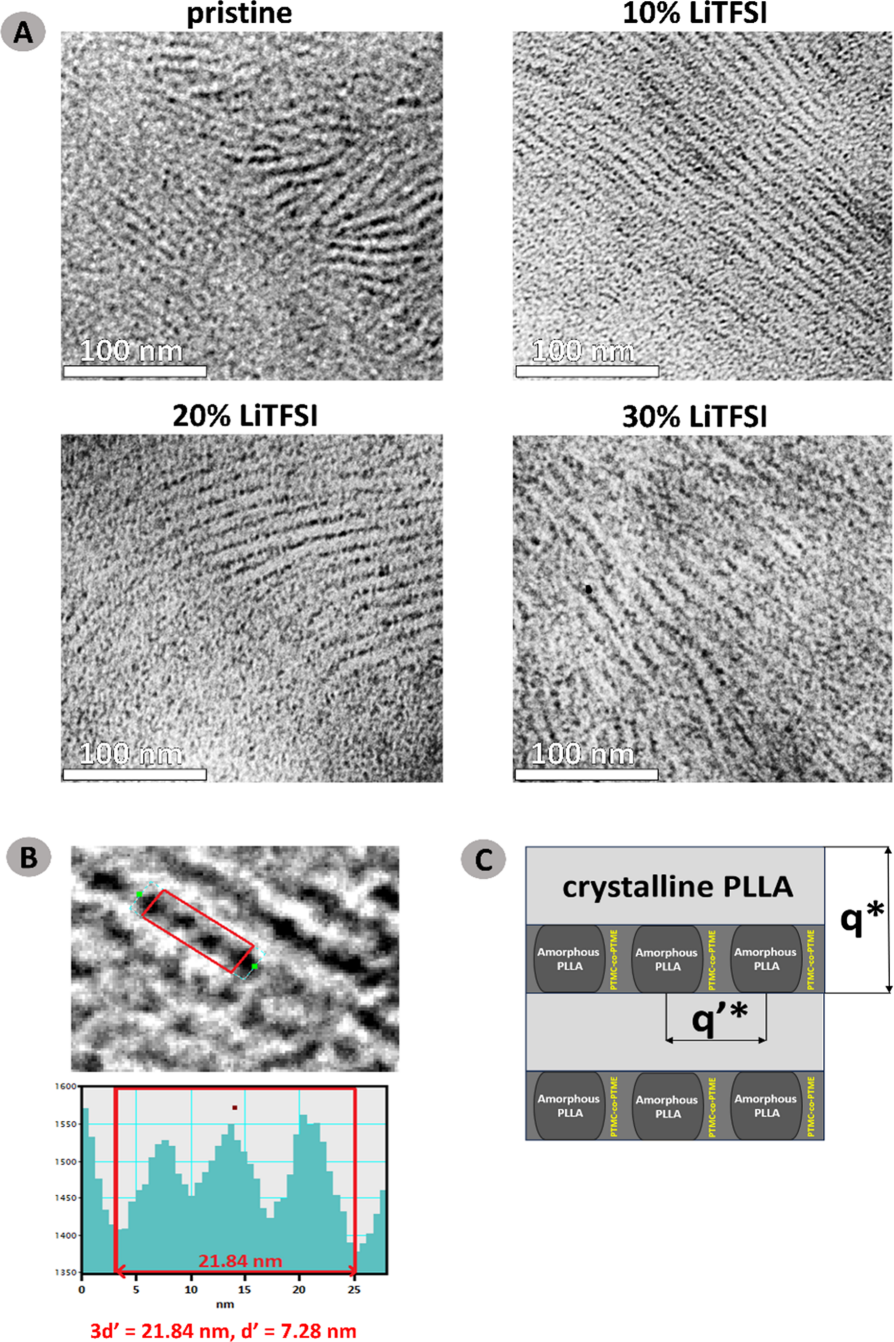
Nanoscale hierarchical morphology of PLLA-b-PTMC-co-PTME-b-PLLA: (a) bright-field TEM micrographs depicting pristine, 10, 20, and 30 wt % of LiTFSI salt-doped BCPs; (b) close-up of 10% LiTFSI micrograph with the estimation of spacing between the separated amorphous domains of PLLA, denoted *d* ^′^; (c) scheme depicting the observed microstructure.

### 3.6 Polarized Light Optical Microscopy

The BCPs were investigated by means of POM where micrographs were recorded as time-lapses during the crystallization process. Samples were crystallized nonisothermally at 100 °C. The influence of the salt content on the superstructure’s morphology, the spherulitic growth rate, and the density of nucleation sites was assessed. Figure 8 reveals the nature of the observed microstructures. As described in the previous sections, crystallization of PLLA led to the formation of nanoscale lamellas, layered with amorphous regions of PTMC-*co*-PTME and PLLA. These lamellae grow radially, resulting in the development of impinged spherulitic superstructures. For the pristine and 10% LiTFSI BCPs, typical Maltese-cross spherulites can be observed. After a critical value of LiTFSI doping (20 wt %), the appearance of superstructures was shifted, where rather irregular extinction bands can be seen.

**Figure 8:**
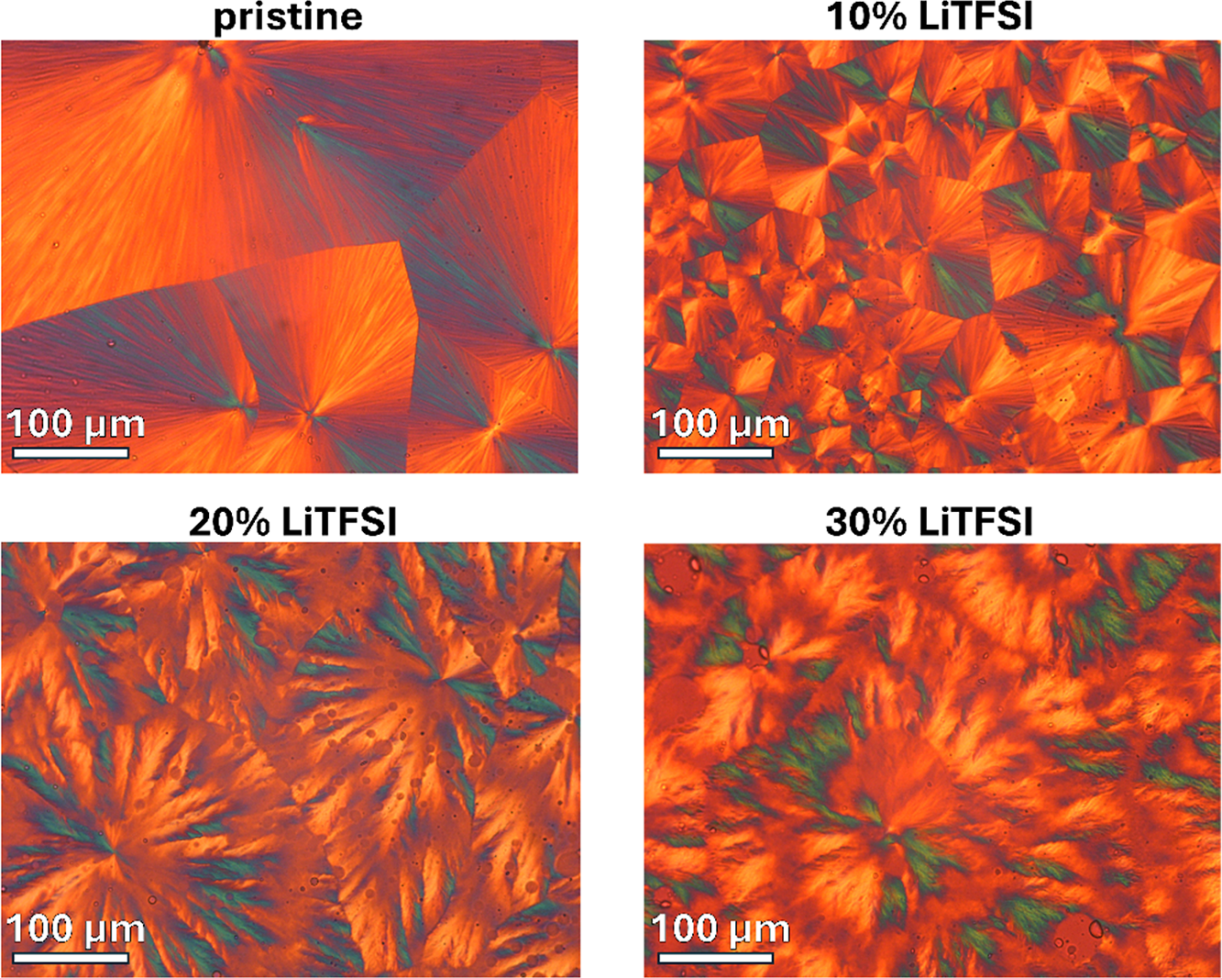
POM micrographs depicting spherulitic superstructures formed by pristine and salt-doped (10 wt %, 20 wt %, and 30 wt %) PLLA-*b*-PTMC-*co*-PTME-*b*-PLLA, crystallized nonisothermally at 100 °C.

The spherulitic growth rate was estimated to be approximately 0.15 μm/s for pristine, 0.06 μm/s for 10% LiTFSI, 0.01 μm/s for 20% LiTFSI, and 0.01 μm/s for 30% LiTFSI. This clearly indicates that salt doping has significantly decreased the growth rate of the crystals. In Figure S8, available in the Supporting Information, the same investigated areas are depicted, where micrographs were captured after 30 min of crystallization time. 10% LiTFSI exhibits higher nucleation density in comparison to that of pristine BCP, suggesting that LiTFSI can act as a nucleating agent for PLLA containing SPEs. Similarly, 20% LiTFSI seems to have slightly higher nucleation density than pristine BCP. However, only one spherulite can be observed on the micrograph depicting 30% LiTFSI, indicating a decrease in the nucleation density upon higher salt loading.

### 3.7 Ionic Conductivity

Figure 9 shows the ionic conductivity of PLLA-*b*-PTMC-*co*-PTME-*b*-PLLA electrolytes with 20 and 30 wt % LiTFSI. The electrochemical impedance measurements were performed in a range 30–150 °C. As confirmed by the DSC and WAXS investigations, the prepared electrolytes contain a PLLA crystalline phase, which is present across the range from room temperature to 100 °C.

**Figure 9:**
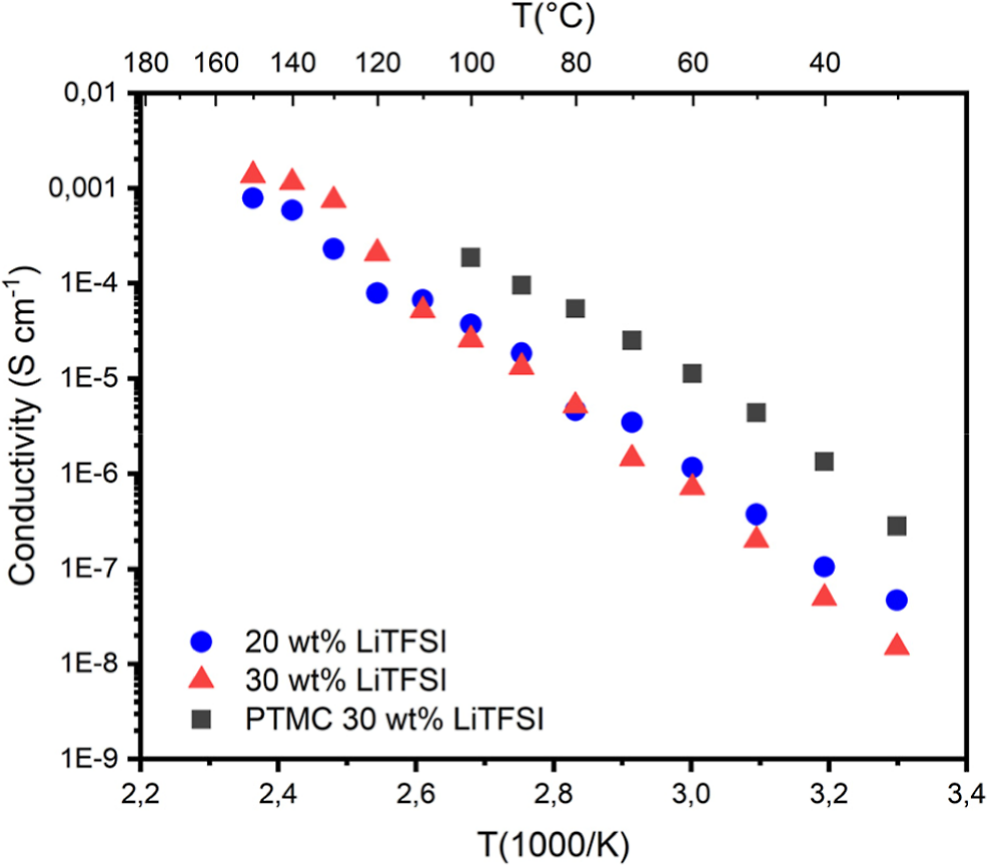
Ionic conductivity measurements of PLLA-b-PTMC-co-PTME-b-PLLA films with different LiTFSI concentrations and PTMC homopolymer with 30 wt % LiTFSI.

Below 100 °C, it is assumed that the salt is dissolved primarily in the PTMC-*co*-PTME-rich phase, as the melting point of PLLA is higher (in the range of 100–140 °C, as seen in DSC). Hence, we attribute the observed conductivity values to the PTMC-*co*-PTME phase. As we focus only on the BCP electrolytes in Figure 9, below 100 °C, the electrolyte with 20 wt % Li salt has slightly higher ionic conductivity. This electrolyte also has a higher degree of crystallinity, so we believe that the salt is pushed to the amorphous phase, which has a smaller volume fraction versus the one with 30 wt % salt, and thus increases the relative salt concentration in the conductive area.

Since there is no pronounced change in conductivity once the melting of the crystallites begins, we attribute the conduction of ions to the PTMC-*co*-PTME-rich phase, regardless of crystalline PLLA being present or not. A slight change in the curve’s slope is observed above 100 °C, but instead of indicating that some of the PLLA contributes to ion conduction, we believe this confirms that the conductivity is dependent on the salt concentration. When a crystalline PLLA phase is present, the salt is concentrated in a smaller portion of the material, only in the amorphous phase; however, above *T*_m_, once the material is completely molten and fully mixed, there will be a dilution of the salt. At this point, the content of salt will have an impact on the conductivity, although slight, which we see clearly in the graph, where at 110 °C, both compositions (20 and 30 wt % LiTFSI) have roughly the same conductivity, but from ≥120 °C, the electrolyte with 30 wt % LiTFSI shows higher values.

To provide a comparison, an electrolyte was also prepared from a previously synthesized PTMC (*M*_n_ = 24,700 g mol^–1^) with 30 wt % LiTFSI. We observe a slight decrease in conductivity at 60 °C for the BCPs compared to the PTMC homopolymer reference (1.1 × 10^–5^ S cm^–1^) with reductions to 1.2 × 10^–6^ S cm^–1^ and 7.2 × 10^–7^ S cm^–1^ at 20 and 30 wt % LiTFSI, respectively. However, a direct comparison cannot be made since the PTMC homopolymer is fully conductive, giving free movement of the ions through the whole volume, whereas the ions are restricted to only the amorphous region in the BCPs, which, as mentioned previously, would be roughly 50 wt % of the whole material. Sun et al.^8^ reported a PTMC electrolyte containing 26 wt % LiTFSI with ionic conductivity in the order of 10^–7^ S cm^–1^ at 60 °C, which is close to the values of our BCP electrolytes, but there may well be an effect of the molecular weight of their material being much higher, *M*_n_ = 368,000 g/mol.

When compared to the PTMC homopolymer, decreased ionic conductivity is expected due to the conducting phase (PTMC-*co*-PTME-rich phase) being reduced. However, the presence of a crystalline phase only slightly affected the conductivity. Considering that the PTMC-based electrolyte completely comprises a conductive material, as opposed to the block copolymeric system, where there is a reduced conductive phase combined with a crystalline phase and where we assume no conduction occurs, the performance of the SPEs here reported is not to be dismissed.

## 4 Conclusions

In this work, SPEs were designed by successfully synthesizing a PLLA-*b*-PTMC-*co*-PTME-*b*-PLLA BCP consisting of a highly crystalline phase and an amorphous conductive phase. We studied the effect of salt concentration on morphology, crystallization rates, and conductivity. It was observed by DSC that PTMC-*co*-PTME and PLLA are partially miscible in the presence of LiTFSI. We confirm that below *T*_m_, PLLA crystals are always present, and the salt dissolves preferably in the PTMC-*co*-PTME-rich phase. We examined how the thermal protocol used in this work influences the self-assembly, crystallization kinetics, and crystalline phase composition via SAXS and WAXS. It was found that adding LiTFSI slows down the crystallization rate and simultaneously alters the ratio of polymorphic forms of PLLA. Through microscopic investigation, we observed multilength-scale hierarchical morphology, where spherulitic superstructures are composed of crystalline PLLA lamellas intertwined with a PLLA/PTMC-*co*-PTME amorphous phase. In the amorphous region, the PLLA phase separated from PTMC-*co*-PTME in the form of nanodomains. This segregation was prompted by the crystallization of PLLA.

Due to the high *T*_m_ of PLLA, the electrolytes could be used at high temperatures, widening the operational temperature window. Ionic conductivities on the order of 10^–6^ S cm^–1^ at 60 °C and 10^–5^ S cm^–1^ at 100 °C were obtained, showing that crystallinity is not a major limit to performance below the melting point. As the addition of PLLA to the structure shows promise for potentially increasing the mechanical properties of formed SPEs, the systematic thermal investigation allowed us to estimate the crystallization rates at the given crystallization intervals. For example, to benefit from the relatively high crystallinity content (above 30%) at elevated salt doping (30 wt %), at least 24 h of crystallization at 100 °C is required. On the other hand, 20% LiTFSI required only 1 h of crystallization to achieve similar crystalline fraction contents while showing higher ionic conductivity below the melting point. Additionally, this polymeric system is fully biodegradable and from renewable sources as compared to more commonly studied BCP for SPEs (i.e., with polystyrene).

This research enhances our understanding of the relationship between microstructure, phase behavior, and ionic transport in polymer electrolytes, informing the design of next-generation energy storage materials.

## Supplementary Material



## Notes

^1^H NMR spectra of the PTMC-*co*-PTME copolymer; additional POM micrographs of BCPs, WAXS, and simultaneous time-resolved SAXS-WAXS spectra of BCPs; and investigation of staining sites (TEM micrograph of PLLA:PTMC-*co*-PTME blend) (PDF)
