# Supplementary material for: Investigating Crystallization and Morphology of PLLA/PTMC Triblock Copolymer Solid Electrolytes
Source: Macromolecules. 2025 Oct 30;58(21):11938–51. doi: 10.1021/acs.macromol.5c02216 (PMC12613804; doi:10.1021/acs.macromol.5c02216)
Supplement: Supplementary file 1 [file ma5c02216_si_001.pdf]

Investigating Crystallization and Morphology of PLLA/PTMC Triblock Copolymer Solid Electrolytes

Adriana Saldívar-Martínez[1,#], Monika Król[2,#], Janne Ruokolainen[2], Tim Melander Bowden[1,*]

1 Department of Chemistry – Ångström Laboratory, Division of Macromolecular Chemistry, Box 538 Uppsala University, Uppsala, Sweden

2 Department of Applied Physics, School of Science, Aalto University, Espoo FIN 00076, Finland

[#] These authors contributed equally to this work

** Email: tim.bowden@kemi.uu.se*

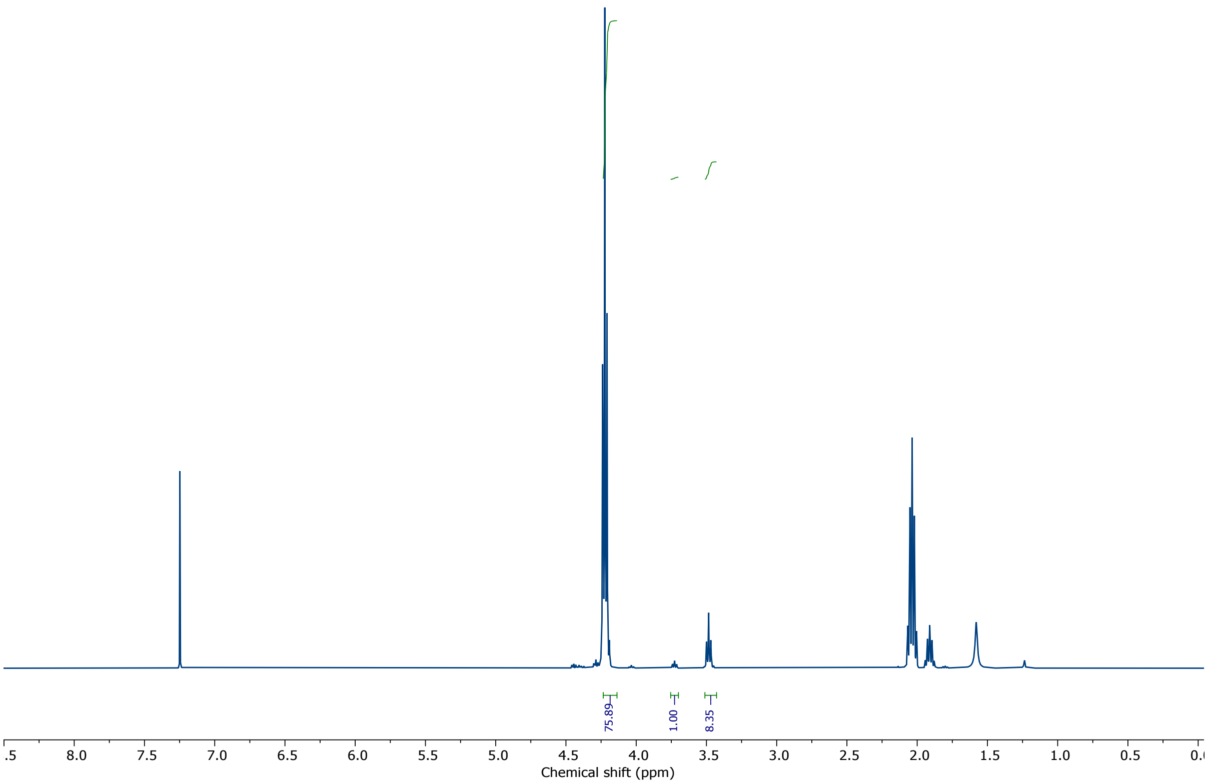

Figure S1. ^{1}H-NMR spectra of PTMC-co-PTME recorded in $CDCl_3$ that appears at 7.2 ppm

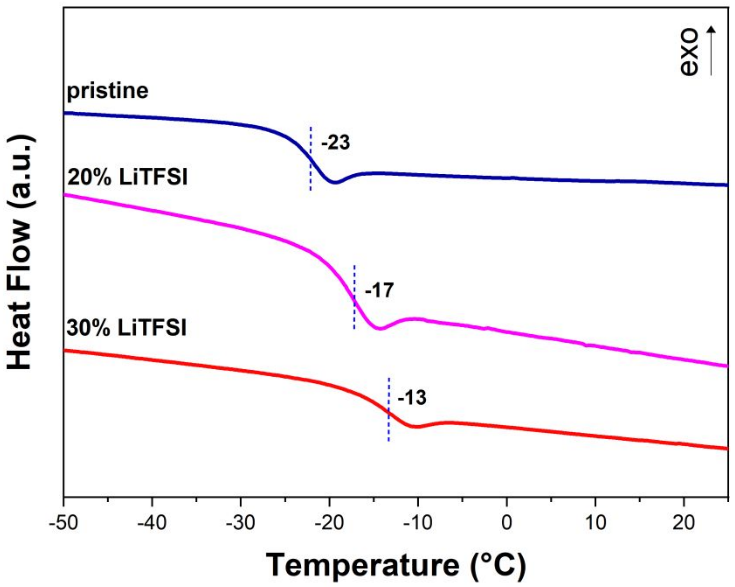

Figure S2. Differential scanning calorimetry (DSC) of PTMC-co-PTME with varying LiTFSI content.

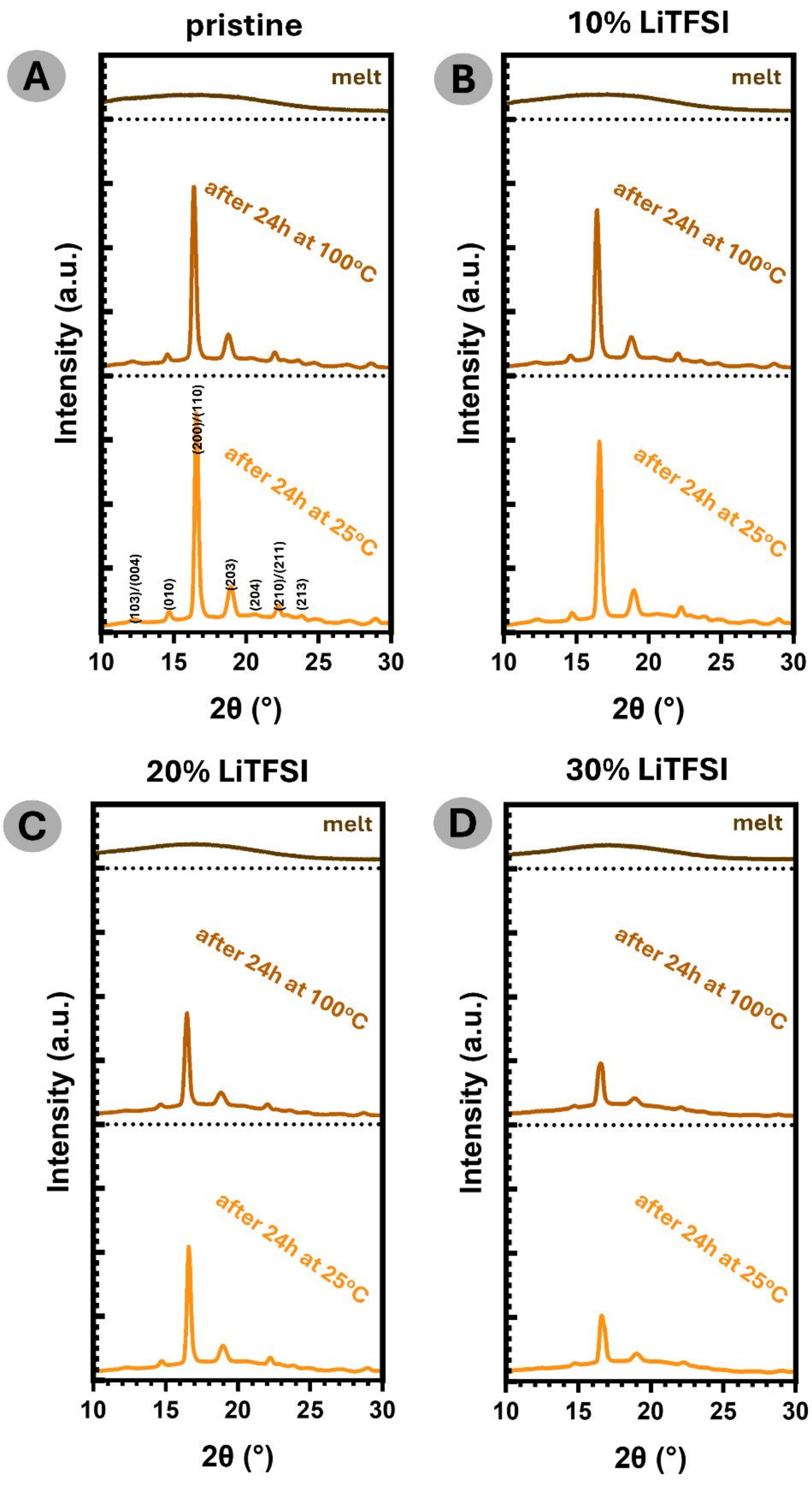

Figure S3. Temperature depended WAXS profiles of a) pristine; b) doped with 10 wt% LiTFSI; c) doped with 20 wt% LiTFSI; d) doped with 30 wt% LiTFSI PLLA-b-PTMC-co-PTME-b-PLLA.

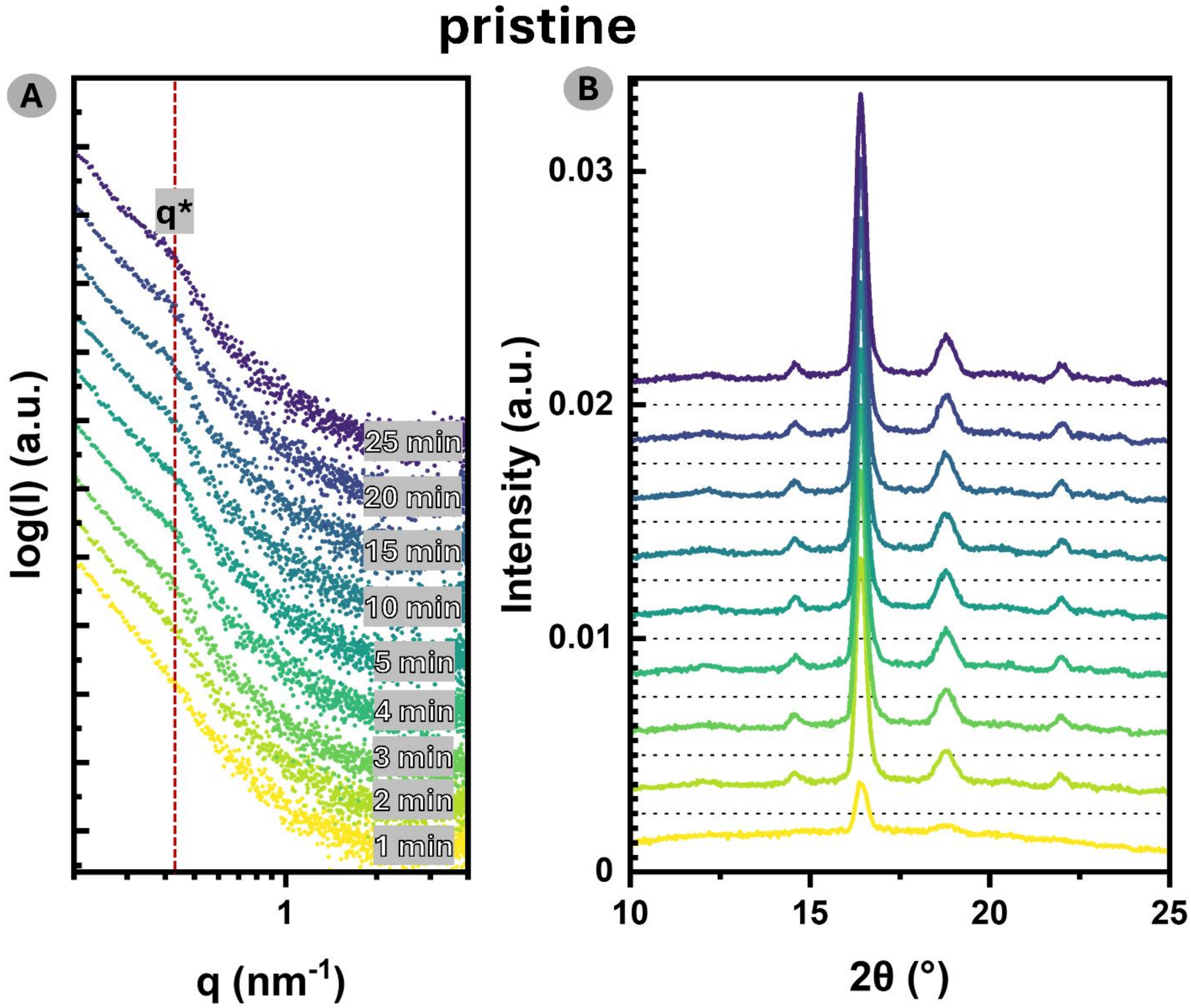

Figure S4. Time-dependent simultaneous SAXS-WAXS spectra for pristine PLLA-b-PTMC-co-PTME-b-PLLA. Sample was non-isothermally crystallized at 100 °C. Timestamps are indicated on the figure.

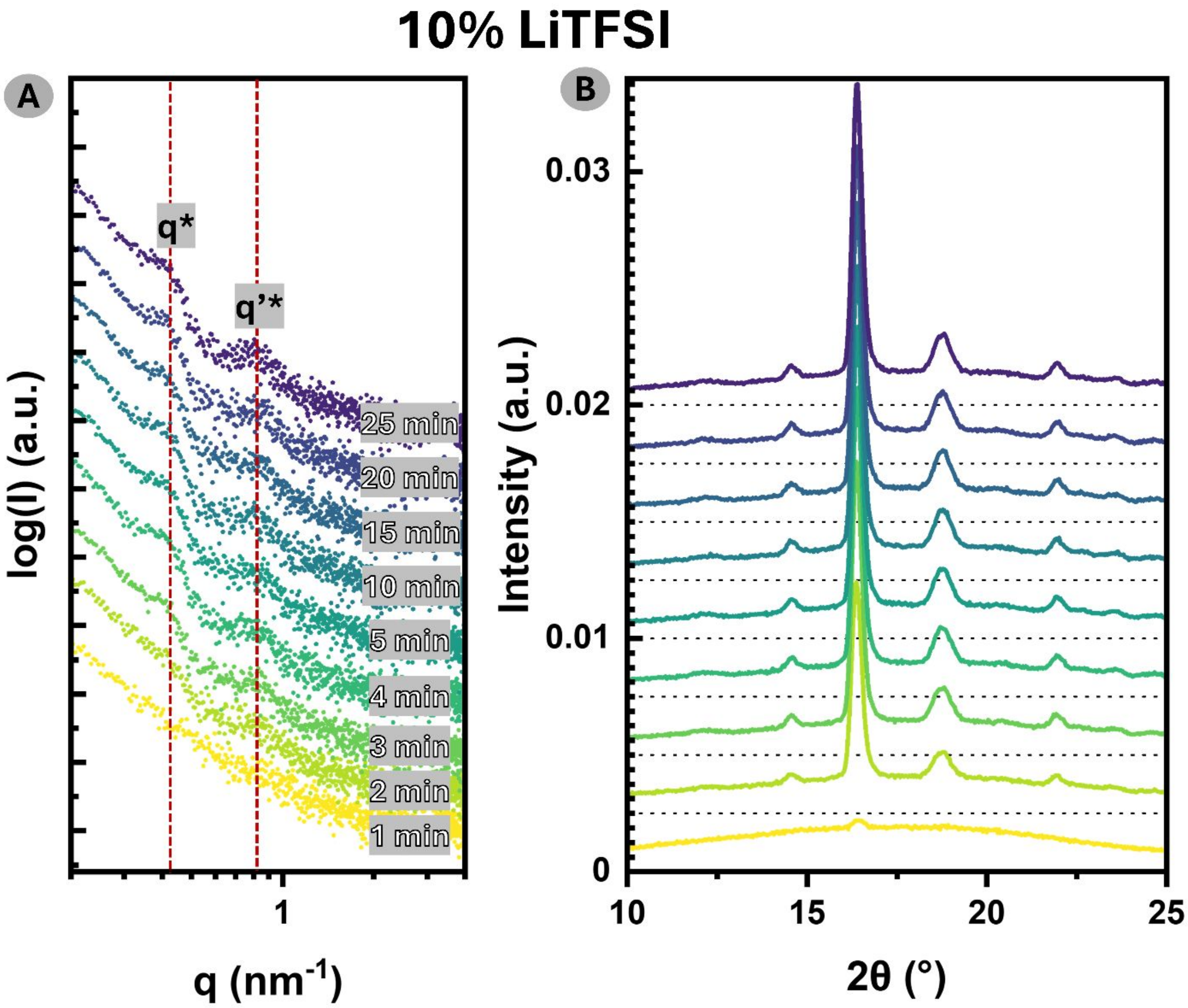

Figure S5. Time-dependent simultaneous SAXS-WAXS spectra for PLLA-b-PTMC-co-PTME-b-PLLA doped with 10 wt% of LiTFSI. Sample was non-isothermally crystallized at 100 °C. Timestamps are indicated on the figure.

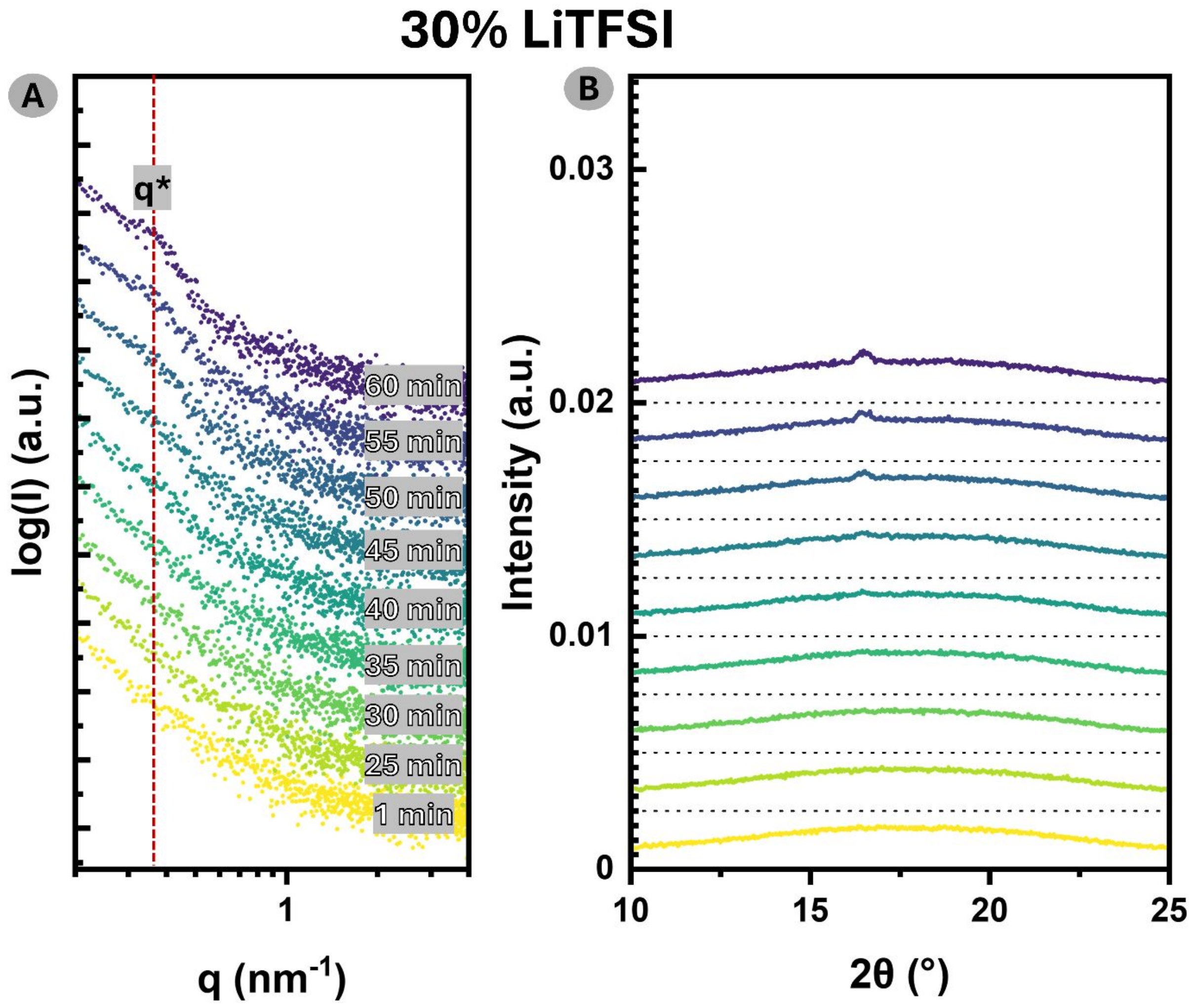

Figure S6. Time-dependent simultaneous SAXS-WAXS spectra for PLLA-b-PTMC-co-PTME-b-PLLA doped with 30 wt% of LiTFSI. Sample was non-isothermally crystallized at 100 °C. Timestamps are indicated on the figure.

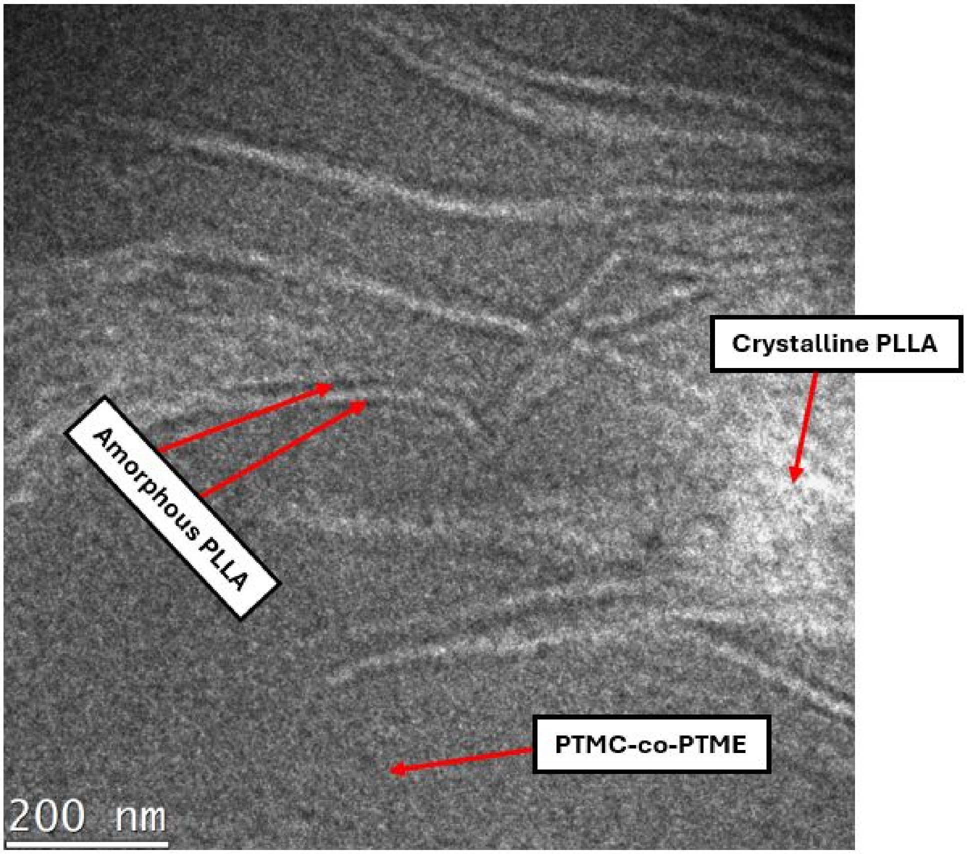

Figure S7. Bright-field TEM micrograph depicting blend with 80:20 w/w PTMC-co-PTME:PLLA. The blend was stained for 10 minutes with RuO_4 vapor. In white, crystalline PLLA; in gray, amorphous PTMC-co-PTME and in dark gray, amorphous PLLA. Higher contrast of amorphous PLLA than amorphous PTMC-co-PTME indicates that it is being stained preferentially by RuO_4.

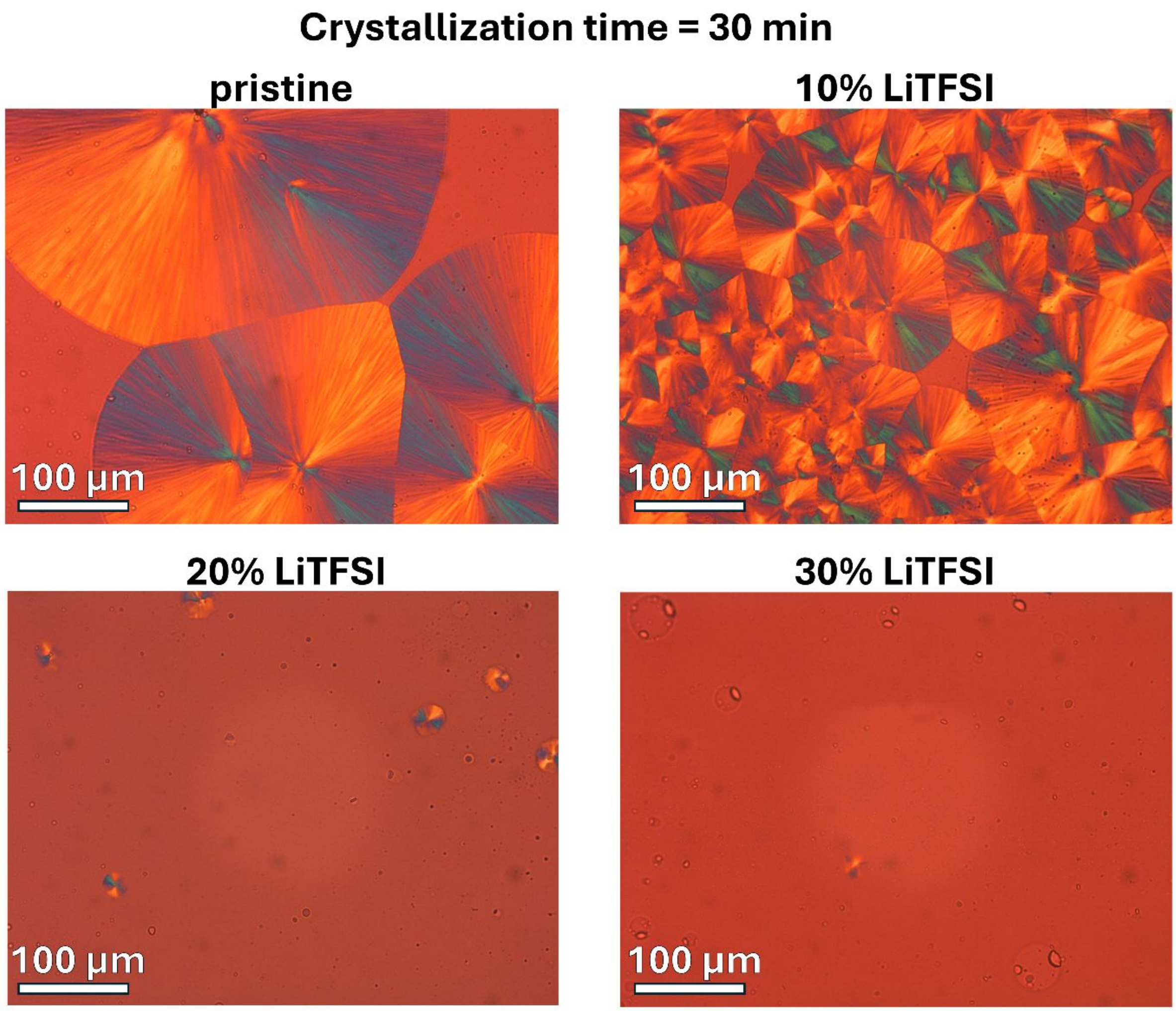

Figure S8. Micrographs depicting spherulitic superstructure of PLLA-b-PTMC-co-PTME-b-PLLA BCPs: pristine, and salt doped (10 wt%, 20 wt%, 30 wt%), captured after 30 min of crystallization at 100 °C.
